# The Impacts of Herbal Medicines and Natural Products on Regulating the Hepatic Lipid Metabolism

**DOI:** 10.3389/fphar.2020.00351

**Published:** 2020-03-24

**Authors:** Sha Li, Yu Xu, Wei Guo, Feiyu Chen, Cheng Zhang, Hor Yue Tan, Ning Wang, Yibin Feng

**Affiliations:** School of Chinese Medicine, Li Ka Shing Faculty of Medicine, The University of Hong Kong, Hong Kong, Hong Kong

**Keywords:** herbal medicines, natural products, lipid metabolism, fatty liver, lipolysis, lipogenesis

## Abstract

The dysregulation of hepatic lipid metabolism is one of the hallmarks in many liver diseases including alcoholic liver diseases (ALD) and non-alcoholic fatty liver diseases (NAFLD). Hepatic inflammation, lipoperoxidative stress as well as the imbalance between lipid availability and lipid disposal, are direct causes of liver steatosis. The application of herbal medicines with anti-oxidative stress and lipid-balancing properties has been extensively attempted as pharmaceutical intervention for liver disorders in experimental and clinical studies. Although the molecular mechanisms underlying their hepatoprotective effects warrant further exploration, increasing evidence demonstrated that many herbal medicines are involved in regulating lipid accumulation processes including hepatic lipolytic and lipogenic pathways, such as mitochondrial and peroxisomal β-oxidation, the secretion of very low density lipoprotein (VLDL), the non-esterified fatty acid (NEFA) uptake, and some vital hepatic lipogenic enzymes. Therefore, in this review, the pathways or crucial mediators participated in the dysregulation of hepatic lipid metabolism are systematically summarized, followed by the current evidences and advances in the positive impacts of herbal medicines and natural products on the lipid metabolism pathways are detailed. Furthermore, several herbal formulas, herbs or herbal derivatives, such as Erchen Dection, Danshen, resveratrol, and berberine, which have been extensively studied for their promising potential in mediating lipid metabolism, are particularly highlighted in this review.

## Introduction

**Figure 1:**
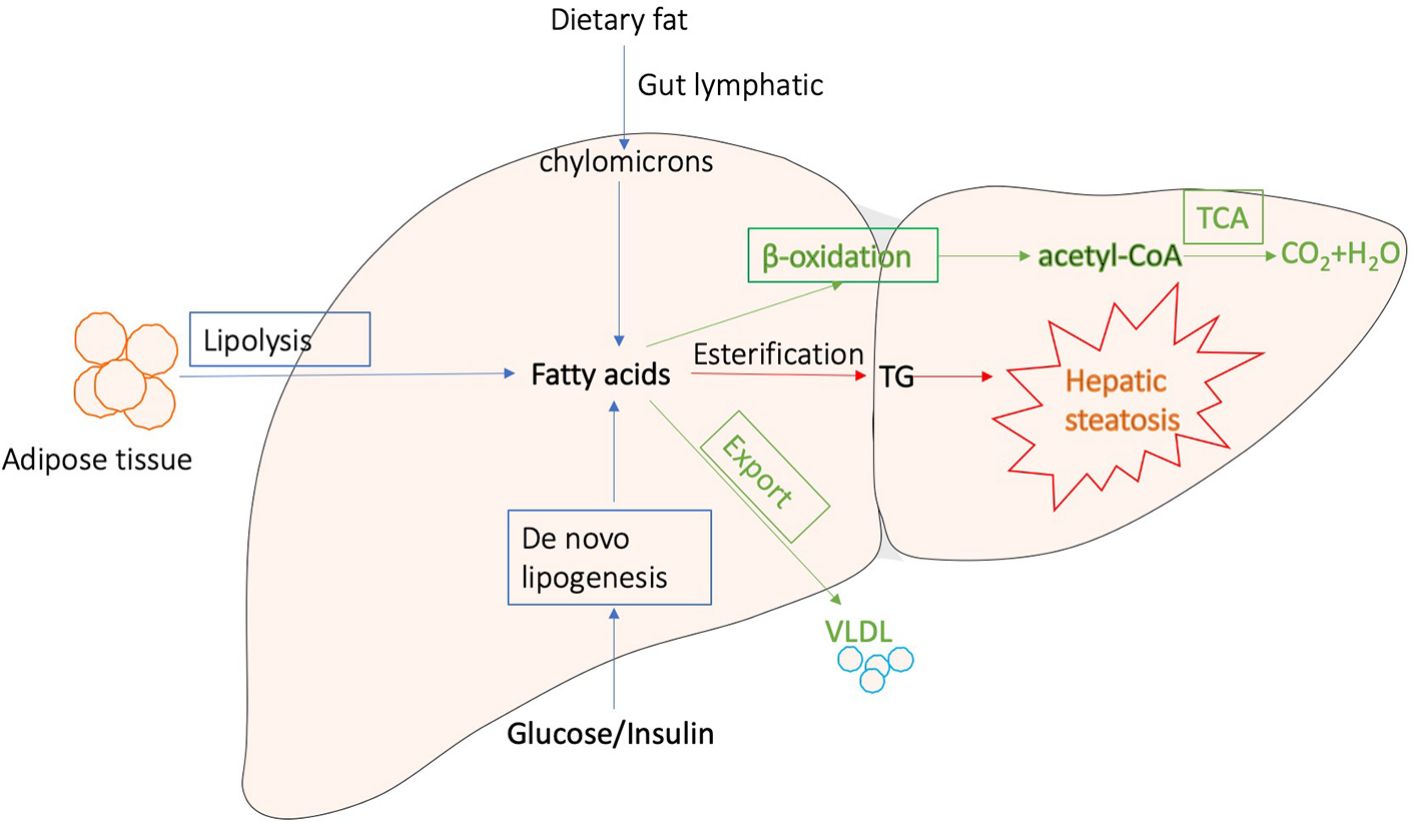
Major processes involved in hepatic lipid metabolism.

Generally, liver regulates lipid metabolism by three major processes: (1) uptake free fatty acids from circulation, and *de novo* fatty acid synthesis (FAS); (2) lipid storage, including converting fatty acids into triglyceride (TG) and other lipid droplets, which are subsequently exported to adipose tissue or stored in liver; and (3) lipid consumption, including lipolysis, β-oxidation, and the generation of lipoproteins (Reddy and Rao, 2006; Musso et al., 2009; Ponziani et al., 2015; Mato et al., 2019). These processes are presented in **Figure 1**. Correct control of lipid level is critical for cellular and organismal homeostasis, while interferences with the lipogenic pathways are accompanied with a variety of metabolic syndromes. The disorders of lipid metabolism, such as decreased β‐oxidation, enhanced lipolysis, and secretion of very low-density lipoprotein (VLDL), as well as altered pathways involved in the FAS, drive the accumulation of lipid droplets into the hepatocytes, eventually leading to the development of hepatic steatosis, which is a common pathological feature in various liver diseases (Reddy and Rao, 2006; Nguyen et al., 2008; Tessari et al., 2009; Perla et al., 2017).

The most prevalent liver diseases resulting from lipid metabolism disorder are alcoholic and non-alcoholic fatty liver diseases. Except difference in alcohol consumption, alcoholic and non-alcoholic fatty liver diseases show similar pathological process, which is characterized by long-term excessive fat accumulation in the liver (Younossi, 2019). They represent a wide range of liver injury, from simple fatty liver through steatosis with necrosis and inflammation to fibrosis and cirrhosis (Lomonaco et al., 2013; Heeboll et al., 2018). In particular, non-alcoholic fatty liver diseases (NAFLD), as the metabolic diseases induced by obesity and type 2 diabetes mellitus, are the second leading causes of death globally, becoming a heavy economic burden in many countries due to the high prevalence (Albhaisi and Sanyal, 2018; Al-Dayyat et al., 2018). Since inordinate lipid metabolism is intensively involved in fatty liver diseases progression, reducing lipid accumulation is a major target of development of pharmaceutical agents for various liver diseases (Ipsen et al., 2018). Simvastatin has been used as lipid-lowing drug in patients with hyperlipidemia (Aronow, 2006). However, it shows side effects, such as constipation headaches, nausea, myopathy, elevated blood sugar, and even liver damage. As a matter of fact, there is currently no satisfying therapeutic drug for fatty liver diseases (Issa et al., 2018; Moctezuma-Velazquez, 2018).

Over the past decades, due to the positive efficacy and minimal side effects, herbal medicines, and natural products have obtained increasing attention as alternative therapeutic agents for liver disorders and dyslipidemia (Xiao et al., 2013; Yao et al., 2016; Liu Q. et al., 2017). Growing evidence from preclinical studies suggests that many herbs and isolated compounds could inhibit the progression of hepatic steatosis (Dong et al., 2012; Liu Z. L. et al., 2013). A variety of mechanisms have been demonstrated to be implicated in preventing hepatic steatosis, including reducing lipogenesis, enhancing β-oxidation, increasing insulin sensitivity, suppressing oxidative stress, and inhibiting activation of inflammatory pathways (Dong et al., 2012; Yao et al., 2016). In recent studies, sterol regulatory elementbinding protein 1c (SREBP-1c), peroxisome proliferator activated receptor α (PPARα), AMP-activated protein kinase (AMPK) and sirtuin 1 (SIRT1) signaling pathways have been highlighted as crucial molecular targets of action mechanisms by which herbal medicines regulate hepatic lipid metabolism (Liu Z. L. et al., 2013). In this review, herbal medicines involved in regulating hepatic lipolytic and lipogenic pathways, such as mitochondrial and peroxisomal β-oxidation, the secretion of very low-density lipoprotein (VLDL), the non-esterified fatty acid (NEFA) uptake, and some vital hepatic lipogenic enzymes are summarized. Current clinical evidences and meta-analysis in the positive impacts of herbal medicines on the hepatic lipid metabolism pathways have also been reviewed. Furthermore, several herbal formulae, herbs or herbal derivatives, such as Erchen Dection, Danshen, resveratrol, and berberine which have been extensively studied for their promising potential in mediating lipid metabolism, are particularly highlighted in this review. This review aims to update and summarize current evidence from laboratory and clinic studies to provide alternative and complementary medical therapies with the regulatory property of hepatic lipid metabolism to current pharmaceuticals for the treatment of liver diseases.

## Herbal Medicines and Natural Products Regulate on the Hepatic Lipid Metabolism Pathways

Increasing evidence indicated that many herbs, natural products, and their derived compounds could inhibit the progression of hepatic steatosis. A variety of mechanisms have been demonstrated to be implicated in preventing hepatic steatosis and modulating lipid metabolism by herbs, including anti-oxidative stress, anti-inflammation, reducing hepatocyte fatty acid uptake and trafficking, reducing hepatic *de novo* lipogenesis, increasing lipolysis, induction of lipophagy, enhancing fatty acid β-oxidation. In particular, SREBP-1c, PPARα, AMPK, and SIRT1 signaling pathways have been highlighted as crucial molecular targets of action mechanisms by which herbal medicines regulate hepatic lipid metabolism. In **Table 1**, we reviewed the effects and mechanisms of herbs and some natural products on fatty liver diseases from recent studies. In the following section, we will discuss herbs that attenuate hepatic steatosis *via* reducing hepatocyte fatty acid uptake and trafficking, reducing hepatic *de novo* lipogenesis, increasing lipolysis, induction of lipophagy, and enhancing fatty acid β-oxidation in detail.

**Table 1:** The effects and mechanisms of herbs and some natural products on fatty liver diseases.

Herbs or Natural products	Model	Effects	Mechanisms	References
*Rosmarinus officinalis* Linn.	Orotic acid induced NAFLD model in rats	Reduced the levels of hepatic TG, TC, FFA and improved cell hypertrophy, vacuolation, and cell necrosis in the liver	↑Phosphorylation of AMPK and ↓SREBP-1c cracking into the nucleus, following ↓FAS	(Wang et al., 2019)
Chinese Herbal Formula (CHF03, composition confidentiality)	HFD induced NAFLD model in mice; AML12 cells treated with palmitic acid *in vitro*	Reduced hepatic steatosis	↓lipogenesis *via* down-regulating the expression of SREBF1, Fasn, and Acaca, ↓ lipid accumulation	(Cui et al., 2019)
Dachaihu Decoction (Bupleuri Radix, Scutellaria baicalensis Georgi, Pinellia ternate, Paeonia lactiflora, Citrus trifoliata, Rheum rhabarbarum, Zingiber officinale, Ziziphus jujuba Mill)	High-fat high-fructose diet induced NAFLD model in rats	Reduced the levels of elevated liver coefficient, serum TG, TC, LDL, AST, and ALT, blood glucose, plasma endotoxin, reduced TG, TNF-α, TGF-β, NF-κB, and TLR4 in liver tissues	↓oxidative stress and inflammation	(Yang J.M. et al., 2019)
Leaves of *Aloysia citrodora* Paláu (syn. Lippia triphylla)	KK‐Ay mice	Improved hepatic lipid metabolism	via activating AMPK	(Zhang Y. et al., 2019)
Polygonatum kingianum	HFD induced NAFLD model in rats	↓ALT, AST, TC, LDL in serum, and hepatic TC and TG	↑mRNA expression of carnitine palmitoyl transferase-1 and ↓uncoupling protein-2 respectively, ↓caspase 9, caspase 3 and Bax expression in hepatocytes, ↑expression of Bcl-2 in hepatocytes and cytchrome c in mitochondria	(Yang X. X. et al., 2019)
Bangpungtongseong-san (Bofutsushosan)	HFD induced NAFLD model in C57BL/6J mice	Ameliorated dyslipidemia and hepatic steatosis, reduced body weight gain	Altered transcriptional changes in the liver, ↓mitochondrial oxidative phosphorylation-related genes in the liver, ↓hepatic fibrosis-related transcriptome.	(Choi et al., 2019)
Thymbra spicata L. extracts	endothelial cells *in vitro*	Ameliorated lipid accumulation, oxidative stress and inflammation, reduced hepatic steatosis	Preventing endothelium dysfunction	(Khalil et al., 2019)
Swertiamarin	fructose-fed mice	Lowed levels of serum glucose, TG, uric acid, ALT, AST, alleviation of hepatic ballooning degeneration and steatosis	↓SREBP-1, FAS and acetyl-CoA carboxylase 1 (ACC1) in liver	(Yang Y. et al., 2019)
Si He Decoction (Zingiber officinale., Cyperus rotundus L., Lilium, Lindera aggregate, Salvia miltiorrhiza, Santalum album, Amomum villosum, *Typha angustifolia* L., Trogopterus xanthipes Milne)	HFD induced NAFLD model in rats	Improved liver pathological conditions	↓expression level of TNF-alpha and IL-6, ↑visfatin, adiponectin, leptin and resistin, targeting adipokines	(Sun et al., 2019)
Modified Longdan Xiegan Tang (composed of *Scutellaria baicalnsis* Geprgi, *Gardenia jasminoides, Adenophora capillaris, Akebia quinate, Plantago asiatica, Angelica sinensis, Rehmannia glutinosa, Alisma plantago-aquatica, Bupleurum gibraltaicum*, and *Glycyrrhiza uralensis*)	Olanzapine-induced fatty liver in rats	↓TG, cell vacuolar degeneration and Oil Red O-stained area	Regulating hepatic *de novo* lipogenesis and fatty acid β-oxidation-associated Gene expression mediated by SREBP-1c, PPAR-α and AMPK-α	(Ren et al., 2019)
LongShengZhi Capsule	apoE-Deficient Mice	Reduced atherosclerosis	↓lipogenic and cholesterol synthetic genes while activating expression of triglyceride catabolism genes	(Ma et al., 2019)
Thymoquinone	Hypothyroidism with NAFLD rats	Reduced steatosis and lobular inflammation	↑antioxidant CAT gene	(Ayuob et al., 2019)
Monomer Hairy Calycosin	NAFLD rats	Control the lipid peroxidation, and reduce the levels of serum TNF-alpha, IL-6, MDA and FFA, improve the steatosis and inflammation of liver tissue	↓CYP2E1, ↓apoptosis of hepatocytes.	(Liu X. et al., 2019)
Hongqi Jiangzhi Formula (Astragali Radix, Red yeast rice, Nelumbinis Folium, Curcumae Longae Rhizoma, Lych Fructus, Magnoliae Officinals Cortex, Artemisiae Scopariae Herba)	HFD induced NAFLD model in rats	Reduced lipid accumulation	↓the expression of NF-kappa B through TLR4 downstream signalling pathways	(Liang et al., 2019)
Jiang Zhi Granule (Herba Gynostemmatis, Folium Nelumbinis, Radix Salviae, Rhizoma Polygoni Cuspidati, and Herba Artemisiae Scopariae)	NAFLD in animal and PA-treated hepatocytes *in vitro*	Showed anti-steatotic effects	droplet degradation *via* autophagy though the mTOR signalling	(Zheng et al., 2018)
Curcumin	Steatotic hepatocyte model *in vitro* and NAFLD rat models	Improved lipid accumulation	Reversed the DNA methylation at the PPAR-alpha gene	(Li Y. Y. et al., 2018)
Samjunghwan Herbal Formula (Mori Fructus, *Lycium chinensis* Miller, Atractylodis Rhizoma)	HepG2 Cells and OLETF Rats	↓Body weights, and visceral adipose tissue (VAT) weights, AST and ALT levels,	↑HMGCOR, SREBP, and ACC, and ↓AMPK and LDLR gene expressions levels.	(Ansari et al., 2018)
Oxyresveratrol	NAFLD in mice	Ameliorated NAFLD	↓LXR alpha agonists-mediated SREBP-1c induction and expression of the lipogenic genes, ↑mRNA of fatty acid beta-oxidation-related genes in hepatocytes; induced AMPK activation, helped inhibit SREBP-1c using compound C.	(Lee et al., 2018)
Sedum sarmentosum Bunge extract	Tilapia fatty liver model	Restored the changes to feed coefficient, immune capacity, and pathological characters	Altered expression of genes in the lipid metabolic process, metabolic process, and oxidation-reduction process. Our results suggest that disorders of the PPAR and p53 signaling pathways	(Huang et al., 2018)
Berberine and curcumin	HFD induced NAFLD model in rats	↓LDL-c, ALT, AST, ALP, MDA, LSP	↓SREBP-1c, pERK, TNF-alpha, and pJNK	(Feng et al., 2018)
Gegen Qinlian decoction (Pueraria lacei Craib, Scutellaria baicalensis Georgi, Coptis chinensis Franch., and Glycyrrhiza uralensis Fisch.) and resveratrol	Rat model of HFD-induced NAFLD	Restored lipid metabolism and inflammatory and histological abnormalities	Triggering the Sirt1 pathway	(Guo et al., 2017)
Gegenqinlian Decoction	Rat model of HFD-induced NAFLD and HepG2	Suppress inflammation and regulate lipid	Improving PPAR-γ	(Wang Y. L. et al., 2015)
Lingguizhugan Decoction (Poria, Ramulus Cinnamomi, Rhizoma Atractylodis Macrocephalae, and Radix Glycyrrhizae)	Rat model of HFD-induced NAFLD	Attenuated phenotypic characteristics of NAFLD	By affecting insulin resistance and lipid metabolism related pathways (e.g., PI3K-Akt, AMPK); activating cholesterol secretio; increasing serum thyroid hormone levels, improving beta-oxidation (via modulation of TR beta 1 and CPT1A expression), metabolism and transport (through modulation of SREBP-1c, ACSL and ApoB100 expression) of fatty acid.	(Liu X. et al., 2017; Yang et al., 2017; Zhu et al., 2017)
Chinese herb extract, QSHX (*Bupleurum falcatum*, *Salvia miltiorrhiza*, rhubarb, lotus leaf, capillary Artemisia, rhizome polygoni cuspidate and gyn*ostemma pentaphyllum*)	High-fat and high-sugar diet-induced NAFLD in rat	↓Body weight, liver index, and serum levels of AST, ALT and TG; and increased the serum level of adiponectin	Promoting the expression of HMW APN and DsbA-L, which may have been induced by inhibiting the activation and expression of FOXO1 in adipocytes	(Liu X. et al., 2017)
Qushi Huayu Decoction (*Herba Artemisiae* capillaris, *Polygonum cuspidatum*, *Hypericum japonicum* Thunb, Gardenia, and *Rhizoma Curcumae* Longae)	NAFLD rats	Attenuated phenotypic characteristics of NAFLD	↑Hepatic anti-oxidative mechanism, ↓hepatic lipid synthesis, and promoted the regulatory T cell inducing microbiota in the gut.	(Feng et al., 2017)
Rhododendron oldhamii Maxim. leaf extract	HepG2 cells and HFD-fed mice	Improves fatty liver syndrome	Increasing lipid oxidation and decreasing the lipogenesis pathway	(Liu Y. L. et al., 2017)
Herbal Formula HT048 (*Crataegus pinnatifida* leaf and *Citrus unshiu* peel extracts.)	HFD-fed rats	Attenuates Diet-Induced Obesity	↓Genes involved in lipogenesis, gluconeogenesis, and adipogenesis, ↑β–oxidation genes	(Lee Y. H. et al., 2016)
Angelica dahurica (Hoffm.) Benth. & Hook.f. ex Franch. & Sav.	HFD-induced hyperlipidemic mice	↓TC and TG in the livers	↓CAT and sterol carrier protein2 (SCP2), ↑ the expression of lipid metabolism related genes-lipase member C (LIPC) and PPAR-γ	(Lu et al., 2016)
Daisaikoto (Bupleuri Radix, Scutellaria baicalensis Georgi, Pinellia ternate, Paeonia lactiflora, Citrus trifoliata, Rheum rhabarbarum, Zingiber officinale, Ziziphus jujuba Mill)	Diabetic fatty liver rats induced by a high-fat diet and streptozotocin (STZ)	Reversing dyslipidemia and insulin resistance	Regulating expressions of SIRT1 and NF-κB	(Qian et al., 2016)
Herb Formula KIOM2012H (*Arctium lappa* Linne, *Glycyrrhiza uralensis* Fischer, *Magnolia officinalis* Rehder & Wilson, Zingiber officinale Roscoe)	HFD-fed mice	Inhibited lipid accumulation	Gene expressions involved in lipogenesis and related regulators	(Park et al., 2015)
Hawthorn (Crataegus) leaf flavonoids	HFD-fed rats	Alleviated NAFLD	Enhancing the adiponectin/AMPK pathway	(Li et al., 2015)
Herbal SGR Formula (Semen Hoveniae extract, *Ginkgo biloba* extract, and *Rosa roxburghii* Tratt extract)	Acute ethanol-induced liver steatosis in mice	Inhibited acute ethanol-induced liver steatosis, ↓serum and hepatic TG level, and improved classic histopathological changes	↓Protein expression of hepatic SREBP-1c and TNF-α and increased adiponectin, PPAR-α, and AMPK phosphorylation in the liver	(Qiu et al., 2015)
Nitraria retusa (Forssk.) Asch. ethanolic extract	db/db mice model	↓Increases in body and fat mass weight, ↓TG and LDL-c levels	↑Gene expression related to lipid homeostasis in liver, modulating the lipolysis-lipogenesis balance	(Zar Kalai et al., 2014)
14-Deoxyandrographolide	Ethanol-induced hepatosteatosis in rats	Alleviate hepatosteatosis	↑AMPK, ↓SREBP-1c, ACC, and FAS, ↑sirtuin I and depletion of malonyl-CoA, ↑fatty acid oxidation	(Mandal et al., 2014)
Total Alkaloids in Rubus aleaefolius Poir	Modified HFD-fed rats	↓TG, TC, and LDL-C levels and ↑HDL-C level	↓Expression of FAS, ACC, ↑carnitine palmitoyltransferase (CPT)	(Li Y. et al., 2014)
Lycium barbarum L. polysaccharide	HFD-fed mice	Improved body compositions and lipid metabolic profiles, ↓hepatic intracellular TG	↓SREBP-1c, ↑AMPK activation	(Li W. et al., 2014)
Salacia oblonga Wall. ex Wight & Arn. root	fructose-induced fatty liver in rats	Diminished fructose-induced fatty liver	↓SREBP-1/1c mRNA and nuclear protein	(Liu L. et al., 2013)
Chunggan extract (*Artemisia capillaries* Thunberg, *Trionyx sinensis* Wiegmann, *Raphanus sativus* Linne, *tractylodes macrocephala* Koidz, *Poria cocos* Wolf, *Alisma orientalis (Sam.)* Juzepczuk, *Atractylodes chinensis* Koidzumi, *Salvia miltiorrhiza* Bunge, *Polyporus umbellatus* Fries, *Poncirus trifoliate* Rafin, *Amomum villosum* Lour, *Glycyrrhiza uralensis* Fisch., *Aucklandia lappa* Decne.)	methionine- and choline-deficient (MCD) diet	↓TG, AST, ALT, ALP, and total bilirubin	Anti-oxidative stress	(Park et al., 2013)
Celastrus orbiculatus Thunb.	HFD-induced NAFLD in guinea pigs	↓TC, free cholesterol (FC), cholesterol ester (CE) and TG in liver	↑mRNA abundance of cholesterol 7 alpha-hydroxylase A1 (CYP7A1) and 3-hydroxy-3-methyl-glutaryl-CoA reductase (HMGCR).	(Zhang et al., 2013)
Oxymatrine	NAFLD rats fed with high fructose diet	↓Body weight gain, liver weight, liver index, dyslipidemia, and TG, ↓liver lipid accumulation.	↓ SREBF1 and ↑PPAR-α	(Shi et al., 2013)
Rhein	HFD-induced obese mice	↓Body weight, particularly body fat content, improved insulin resistance, and ↓circulating cholesterol levels, ↓TG, reversed hepatic steatosis, and normalized ALT	Mediated negative energy balance, metabolic regulatory pathways, and immunomodulatory activities involved in hepatic steatosis	(Sheng et al., 2011)
Osthol	Alcohol-induced fatty liver in mice	Inhibit alcohol-induced fatty liver	Anti-oxidation and suppression of TNF-α production	(Sun et al., 2009)

↑ means increase and up-regulate and ↓ means decrease and down-regulate.

### Reducing Hepatocyte Fatty Acid Uptake and Trafficking

Nonesterified fatty acids (NEFAs) and glycerol are generated and released from adipose tissue *via* lipolysis (Kawano and Cohen, 2013). Then NEFAs enter into hepatocytes principally through CD36, and fatty acid transports (FATPs)(Kawano and Cohen, 2013). Several mediators have been demonstrated to play a role in regulating CD36 and FATPs, such as pregnane X receptor (PXR), which impact the hepatocyte fatty acid uptake. Increasing evidence has shown that a variety of herbs and natural compounds attenuate hepatic steatosis *via* modulating genes for fatty acid uptake.

Scutellarin, one of the Traditional Chinese Medicines (TCM) used for liver diseases and diabetes, was found to reduce insulin-dependent lipid accumulation and the mRNA expression of CD36 in HepG2 cells-treated with palmitic acid (Luan et al., 2019). Several other TCM and isolated compounds, babaodan, licorice extract, polyphenol-enriched fraction from Herba Erigerontis, and magnesium lithospermate B, reduced hepatic CD36 expression in mice fed with High Fat Diet (HFD) (Wu and Wang, 2012; Wang et al., 2016; Sheng et al., 2019). Dansameum reduced the expression level of CD36 in liver of apolipoprotein E-Knockout mice with NAFLD (Ahn et al., 2019). In another mice model of NAFLD induced by high-fat and high-cholesterol diet, gypenosides which are a type of TCM extracted from plants downregulated CD36 level in the liver, alleviating the progression of hepatic steatosis (Huang et al., 2019). Berberine attenuated fat accumulation in the liver partially *via* suppressing the expression of FATP gene in HFD-fed mice (Zhou et al., 2019).

### Reducing Hepatic De Novo Lipogenesis

**Figure 2:**
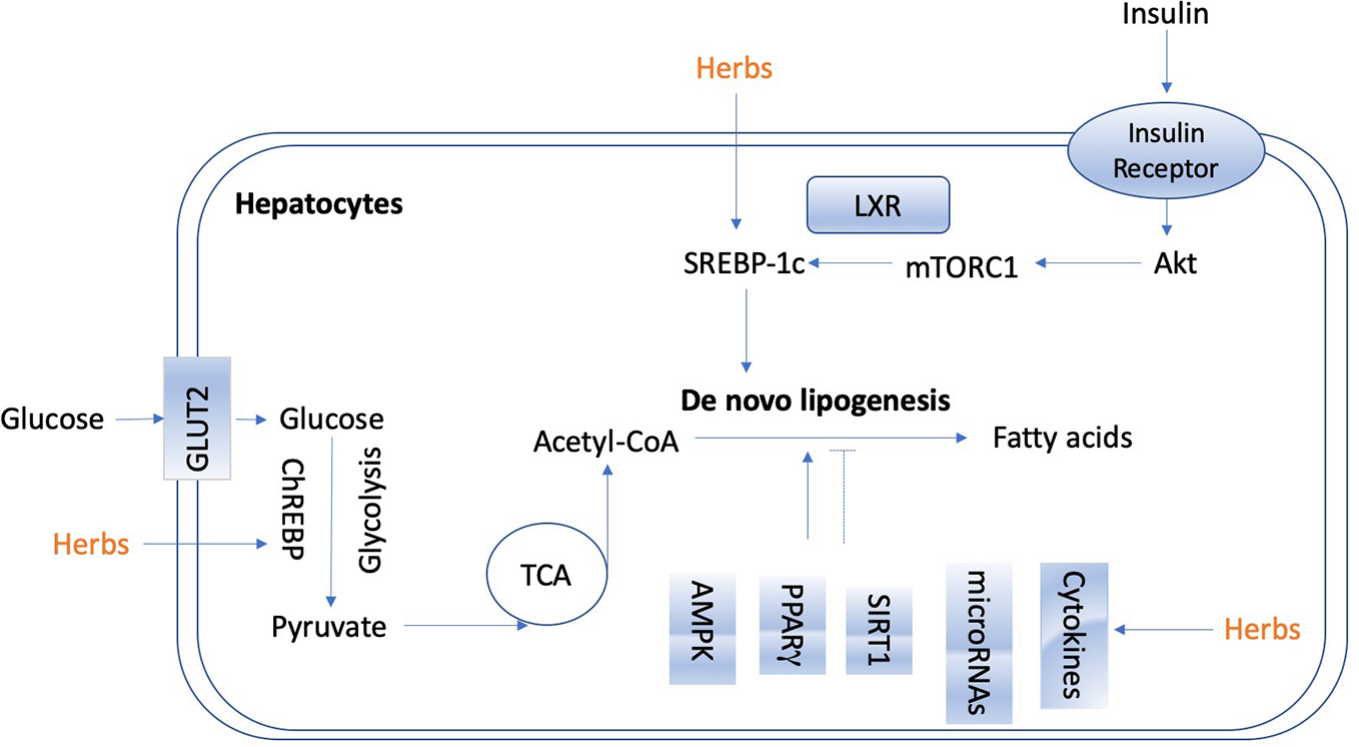
The overview of lipogenesis in hepatocytes.

*De novo* lipogenesis in the liver is tightly controlled by metabolic hormones such as insulin, and glucose level (Wang Y. et al., 2015). In the normal physiological status, high level of glucose promotes the secretion of insulin, activates carbohydrate-responsive element-binding protein (ChREBP), and meanwhile, provides substrate to facilitate lipogenesis in the liver (Wang Y. et al., 2015). In terms of insulin, it activates sterol regulatory element-binding protein 1c (SREBP-1c) to up-regulate lipogenic enzymes, and then promotes *de novo* lipogenesis (Eissing et al., 2013; Chao et al., 2019). **Figure 2** shows the overview of lipogenesis in hepatocytes. Herbs and isolated natural compounds have been demonstrated by animal studies and *in vitro* studies to alleviate hepatic steatosis by ChREBP pathway and insulin-SREBP-1c pathway, as well as other factors, such as AMPK, PPARγ, SIRT1, inflammatory cytokines, immuno-modulation, and microRNAs. We summarized medicinal herbs and isolated natural compounds from recent literatures with the effects of reducing hepatic lipogenesis in **Table 2** and discussed some representative studies in detail as following.

**Table 2:** Medicinal herbs and isolated natural compounds with the effect of hepatic lipogenesis reduction.

Herbs or compounds	Model	Effect	Mechanism	References
Dansameum (*Salvia miltiorrhiza* root)	Apolipoprotein E-Knockout mice	Reduced hepatic lipogenesis and inflammation	Regulating LXR-*α*, PPAR-*γ*, SREBP-1, FAS, ACC1, and CD36	(Ahn et al., 2019)
Alisol A	HFD-induced obese mice	Reduced hepatic steatosis and improved liver function	AMPK/ACC/SREBP-1c pathway	(Ho et al., 2019)
Ling-gui-zhu-gan decoction (*Poria cocos, Ramulus cinnamomi, Atractylodis macrocephalae Rhizoma* and *Radix glycyrrhizae* )	HFD-fed rats	Reduced hepatic glycogen	Inhibited the activity of ACC, SREBP-1c and HMGCR, *via* inhibiting PPP1R3C targeting pathways	(Dang et al., 2019)
Salvianolic acids	Ovariectomized rats	Reduced body weight gain and attenuated	Blocking STAT-3/SREBP1 signaling	(Dang et al., 2019)
Gyeongshingangjeehwan 18 (*Laminaria japonica*, *Rheum palmatum*, and *Ephedra sinica*)	HFD-induced obese mice	Attenuated visceral obesity and NAFLD	Down-regulated lipogenesis-related genes	(Lim et al., 2018)
Cordycepin	Oleic acid-induced mouse FL83B hepatocytes	Attenuated lipid accumulation	Activating AMPK and regulating mitochondrial function	(Uen et al., 2018)
Oxyresveratrol	HFD-fed mice	Ameliorated NAFLD	AMPK/SREBP-1c pathway	(Lee et al., 2018)
Berberine	MIHA and HepG2 cells	Reduced hepatosteatosis	Up-regulation of miR-373 decreased mRNA level target gene AKT1, leading to inhibition of AKT-mTOR-S6K signaling pathway in hepatocytes	(Li C. H. et al., 2018)
Genipin	HFD-fed mice	Reduced HFD-induced hyperlipidemia and hepatic lipid accumulation	Increased the expression levels of miR-142a-5p, which bound to 3 untranslated region of SREBP-1c	(Zhong et al., 2018)
Gangjihwan (*Ephedra intermedia* Schrenk & C.A.Mey., *Lithospermum erythrorhizon* Siebold & Zucc., and *Rheum palmatum* L.)	HFD-induced obese mice	Inhibited fat accumulation	Modulation of lipogenic transcription factors SREBP-1c, PPAR-γand ChREBP-*α*	(Jang et al., 2018)
Gangjihwan (*Ephedra intermedia* Schrenk & C.A.Mey., *Lithospermum erythrorhizon* Siebold & Zucc., and *Rheum palmatum* L.)	HFD-fed C57BL/6 J mice and HepG2 cells	Anti-obesity and anti-nonalcoholic steatohepatosis	Increased mRNA levels of fatty acid oxidation genes and decreased mRNA levels of genes for lipogenesis	(Roh et al., 2017)
Dangguiliuhuang Decoction (root of Rehmannia Glutinosa, *Angelica acutiloba* Siebold et Zucc., *Coptis chinensis* Franch., *Radix Rehmanniae Praeparata*, *Astragalus propinquus*, *Scutellaria baicalensis*, and *Phellodendron chinense* Schneid.)	ob/ob mice	Normalized glucose and insulin level, increased the expression of adiponectin, diminished fat accumulation and lipogenesis, and promoted glucose uptake	↓T cells, ↑ Tregs differentiation, ↓DCs maturation, ↓ DCs-stimulated T cells proliferation and secretion of IL-12p70 cytokine, promoted the interaction of DCs with Tregs, changed PI3K/Akt signaling pathway and↑ PPAR-γ.	(Cao et al., 2017)
Glycycoumarin	MCD diet mice	Prevented hepatic steatosis	Activation of AMPK signaling pathway	(Zhang et al., 2016)
Gambigyeongsinhwan (Curcuma longa, Alnus japonica, and Massa Medicata Fermentata)	Otsuka Long-Evans Tokushima fatty rats and HepG2 cells	Suppressed hepatic steatosis and obesity-related hepatic inflammation	↓mRNA levels of FAS, ACC1, ChREBP alpha, and SREBP-1c	(Yoon et al., 2017)
Alisol B 23-acetate	MCD diet-fed mice	↓ALT, AST, TG	FXR-dependent, ↓hepatic lipogenesis through decreasing hepatic levels of SREBP-1c, FAS, ACC1 and SCD1 and ↑lipid metabolism *via* inducing PPAR α, CPT1 α, ACADS and LPL	(Meng et al., 2017)
Herbal Formula HT048 (*Crataegus pinnatifida* leaf and *Citrus unshiu* peel extracts)	HFD-fed obese rats	Decreased obesity and insulin resistance	↓Genes involved in lipogenesis	(Lee Y. H. et al., 2016)
Jatrorrhizine hydrochloride	HFD-induced obesity mouse model	Attenuated hyperlipidemia	↓ SREBP-1c and FAS, and induced PPAR- and CPT1A	(Yang et al., 2016)
Puerarin	Oleic acid (OA)-treated HepG2 cells	Ameliorated hepatic steatosis	↑PPAR-α and AMPK signaling pathways, ↓SREBP-1 and FAS expression	(Kang et al., 2015)
Protopanaxatriol	HFD-induced obesity (DIO) mice	Alleviated steatosis	Inhibition of PPAR-γ activity	(Zhang et al., 2014)
Magnolia officinalis Rehder & E.H.Wilson	HepG2 cells and mouse normal FL83B hepatocytes	Attenuated TG biosynthesis	Inhibition of SREBP-1c *via* AMPK phosphorylation	(Seo et al., 2014)
Lycium barbarum polysaccharide	HFD-fed mice	Attenuate liver steatosis	↓SREBP-1c expression *via* AMPK activation	(Li W. et al., 2014)
Houttuynia cordata Thunb.	HepG2	Attenuates Lipid Accumulation	AMPK signaling	(Kang and Koppula, 2014)
Berberine metabolites	HepG2	TG-lowering effects	↓Lipogenesis gene expressions through activation of the AMPK signaling pathway	(Cao et al., 2013)
3-Caffeoyl, 4-dihydrocaffeoylquinic acid from *Salicornia herbacea Salicornia europaea* L.	HepG2	Attenuated high glucose-induced hepatic lipogenesis	Prevented lipid accumulation by blocking the expression of SREBP-1c and FAS through LKB1/SIRT1 and AMPK activation	(Pil Hwang et al., 2013)
Fructus Xanthii (*Xanthium strumarium*)	HFD-fed rats	Attenuated hepatic steatosis	↓The expression of lipogenic genes	(Li et al., 2013)

↑ means increase and up-regulate and ↓ means decrease and down-regulate.

Magnolia officinalis Rehder & E.H.Wilson, *Houttuynia cordata* Thunb., 3-Caffeoyl, 4-dihydrocaffeoylquinic acid from *Salicornia europaea* L., puerarin and four kinds metabolites of berberine attenuated lipid accumulation in HepG2 cells *in vitro via* down-regulation of lipogenesis gene expressions through activation of the AMPK signaling pathway (Cao et al., 2013) (Pil Hwang et al., 2013; Kang and Koppula, 2014). Gyeongshingangjeehwan 18 (an herbal drug composed of *Laminaria japonica*, *Rheum palmatum*, and *Ephedra sinica*), Herbal Formula HT048 (*Citrus unshiu* and *Crataegus pinnatifida*), Fructus Xanthii (*Xanthium sibiricum* Patr.), *Lycium barbarum* polysaccharide, Jatrorrhizine hydrochloride, oxyresveratrol, and alisol A isolated from *Rhizoma alismatis* (Oriental Waterplantain Tuber.) attenuated liver steatosis in HFD-fed animals *via* regulating lipogenic genes, predominantly relating with downregulation of SREBP-1c expression *via* AMPK activation (Li et al., 2013; Li W. et al., 2014; Lee Y. H. et al., 2016; Yang et al., 2016; Lee et al., 2018; Lim et al., 2018; Ho et al., 2019). Gangjihwan, a polyherbal composition of *Ephedra intermedia* Schrenk & C.A.Mey., *Lithospermum erythrorhizon* Siebold & Zucc., and *Rheum palmatum* L., showed anti-obesity and anti-nonalcoholic steatohepatosis effects in HFD-fed mice. Lipogenic transcription factors, SREBP-1c, PPAR-γ, and ChREBP alpha were involved in the action mechanism (Jang et al., 2018); (Roh et al., 2017). Molecular targets of FAS, ACC1, ChREBP alpha, and SREBP-1c were also found to be involved in the underlying mechanism of anti-hepatic steatosis and anti-obesity-related hepatic inflammation effect of Gambigyeongsinhwan in Otsuka Long-Evans Tokushima fatty rats and HepG2 cells (Yoon et al., 2017).

Glycycoumarin, a representative of coumarin compounds isolated from licorice, and Alisol B 23-acetate exert ability of reducing hepatic lipogenesis in methionine-choline-deficient (MCD) diet-fed mice (Meng et al., 2017; Zhang E. et al., 2019). MCD diet is a classical dietary model of non-alcoholic steato-hepatitis. With the lack of methionine and choline and high sucrose (40%) and fat (10%), impaired hepatic mitochondrial β-oxidation and very low-density lipoprotein (VLDL) synthesis are observed in mice (Ibrahim et al., 2016). Glycycoumarin activated AMPK signaling pathway to reduce lipogenesis. Alisol B 23-acetate, a natural triterpenoid derived from TCM *Rhizoma alismatis* (Oriental Waterplantain Tuber.), decreased hepatic lipogenesis *via* FXR-dependent pathway. It decreased hepatic levels of SREBP-1c, FAS, ACC1 and SCD1, and promoted lipid metabolism *via* inducing PPAR α, CPT1 α, ACADS, and LPL (Meng et al., 2017). In an apolipoprotein E-knockout mice model, Dansameum (*Salvia miltiorrhiza* root), a kind of Korean polyherbal medicine, reduced hepatic lipogenesis, and inflammation *via* regulating PPAR-γ, SREBP-1c, FAS, ACC1, and CD36 (Ahn et al., 2019).

Dangguiliuhuang Decoction, a TCM formula composed of *radix rehmanniae* (root of Rehmannia Glutinosa), *angelica* (*Angelica acutiloba* Siebold et Zucc.), *Coptis chinensis* Franch., *Radix Rehmanniae Praeparata* (Rehmannia root), *Astragalus propinquus* (the root of *astragalus membranaceus*), Chinese skullcap (*Scutellaria baicalensis*) and *Phellodendron amurense* (*Phellodendron chinense* Schneid.), is used for the treatment of autoimmune diseases and diabetes (Cao et al., 2017; Cao et al., 2018). In a study of ob/ob mice model, it normalized glucose and insulin level, diminished fat accumulation and lipogenesis, increased the expression of adiponectin, and promoted glucose uptake (Cao et al., 2017). It showed modulation abilities on inflammation and immune response. Dangguiliuhuang Decoction (composition as listed above) promoted the shift of pro-inflammatory to anti-inflammatory cytokines. Furthermore, it decreased T cells proliferation while increased regulatory T cells (Tregs) differentiation, reduced dendritic cells (DCs) maturation and secretion of IL-12p70 cytokine, decreased DCs-stimulated T cells proliferation, and promoted, the interaction of DCs with Tregs. In adipocytes and hepatocytes as well as DCs and T cells, Dangguiliuhuang Decoction treatment altered PI3K/Akt signaling pathway and increased PPAR-γ expression, indicating the ameliorated glucose and lipid metabolism (Cao et al., 2017).

MicroRNA (miR), a small non-coding RNA molecule, has been recently demonstrated to play a role in mediating the anti-hepatic steatosis effects of natural compounds derived from herbs. Berberine reduced steatosis in MIHA and HepG2 cells by mechanism associating with up-regulation of miR-373, which decreased its mRNA level target gene AKT serine/threonine kinase 1 (AKT1), resulting in the suppression of AKT-mTOR-S6K signaling pathway in hepatocytes (Cao et al., 2018). Genipin reduced HFD-induced hyperlipidemia and hepatic lipid accumulation in mice *via* increasing the expression levels of miR-142a-5p, which bound to 3’-untranslated region of SREBP-1c, thus leading to the inhibition of lipogenesis (Zhong et al., 2018).

### Increasing Lipolysis

Lipolysis is the catabolic process of hydrolytic cleavage of ester bonds in TG, leading to the production of fatty acids and glycerol, which could be further utilized for β-oxidation and subsequent ATP generation (Lass et al., 2011). It predominantly occurs in adipose tissues, but also in the liver, with different physiological functions. Dietary fat is digested into the gut lymphatic system as chylomicrons, which arrives at the liver through the circulation and release NEFAs through lipolysis which mediated mainly by lipoprotein lipase (LPL) (Rui, 2014). Other lipolytic enzymes contributing to hepatic TG metabolism include adiponutrin/patatin-like phospholipase domain containing 3 (PNPLA3) (Kumashiro et al., 2013), lysosomal acid lipase (LAL) (Quiroga and Lehner, 2018), arylacetamide deacetylase (Lo et al., 2010), hepatic lipase (HL)(Chatterjee and Sparks, 2011) and some members of the carboxylesterase family. In adipose tissue, inhibition of lipolysis improves glucose metabolism and insulin sensitivity, whereas in liver tissue, increasing lipolysis facilitates the attenuation of hepatic steatosis.

As far from now, limited herbs were found to show regulatory effect on hepatic lipolysis. *Lavatera critica* (Cornish mallow), a green leafy vegetable, attenuated hepatic lipid accumulation induced by HFD *via* reversing lipolysis genes acetyl-CoA carboxylase (Veeramani et al., 2017). *Nitraria retusa* (Forssk.) Asch. ethanolic extract modulated the lipolysis-lipogenesis balance in the liver of db/db mice (Veeramani et al., 2017). Caffeic acid upregulated the phosphorylation of AMPK and its primary downstream targeting enzyme, acetyl-CoA carboxylase, to promote the lipolysis in HepG2 cells with oleic acid administration (Liao et al., 2014). *Polygonatum stenophyllum* (PS) Maxim. rhizome showed efficacy on menopausal obesity by activating lipolysis-related genes including hormone-sensitive lipase (HSL) and adipose triglyceride lipase (ATGL) (Lee J. E. et al., 2016). Mulberry (Fructus Mori) water extracts promoted hepatic lipolysis and protected liver from steatosis in obesity (Peng et al., 2011). More herbs or natural compounds exerted effects on lipolysis in adipose tissues and attenuated hepatic steatosis *via* liver-adipose tissue crosstalk, which are not going to be discussed in detail here.

### Induction of Lipophagy

In addition to lipolysis, lipid breakdown can also be accessed *via* lipophagy, a special kind of autophagy to degrade lipid droplets (Singh and Cuervo, 2012; Kounakis et al., 2019). It is a process that the double membrane wraps lipid droplets and sends them to lysosomes to form autolysosomes for degradation of excessive lipid droplets deposited in cells (Liu and Czaja, 2013; Ward et al., 2016). It plays a vital role in maintaining the cellular steady state. During the early stage of NAFLD, lipophagy is activated in response to acute increase in lipid availability, thus reduce lipid deposition (Czaja, 2016; Ipsen et al., 2018). However, in the condition of such as long-lasting high fat dieting, hepatic lipophagy is impaired when lipids are sustained overwhelmed (Kwanten et al., 2014; Czaja, 2016; Ipsen et al., 2018). Growing evidence raised from recent studies indicate that lipophagy is partially suppressed in patients and animal models of NAFLD and restoring lipophagy may slow the progression of hepatic steatosis. Lipophagy could be activated by various approaches, such as mTOR and AMPK-targeting agents. Glycycoumarin, a representative of coumarin compounds isolated from licorice, mitigated hepatic steatosis partially through AMPK-mediated lipophagy in a murine model of NAFLD induced by MCD diet (Zhang et al., 2016). Dioscin is a saponin extracted and isolated from *Polygonatum zanlanscianense* Pamp. It has been proposed as a healthcare product against hepatic fibrosis with remarkable ability to inhibit the expression of p-mTOR/mTOR level and sequentially promote autophagy (Xu et al., 2017). In another study, Bergamot polyphenol fraction prevents NAFLD *via* stimulation of lipophagy in cafeteria dietinduced rat model of metabolic syndrome. The increased levels of LC3 and Beclin 1, and concomitant reduction of SQSTM1/p62 proved the promoted lipophagy with the treatment of Bergamot polyphenol fraction (Parafati et al., 2015). Increasing number of herbs or natural products have been demonstrated to exert significant effects on regulating lipophagy in the liver. Current understanding of mechanisms associated with autophagy/lipophagy of herbal medicines and natural products in preventing and treating NAFLD has been well reviewed in Zhang et al. (2018), which could be referred for further reading.

In alcoholic liver diseases (ALD), upon acute consumption of alcohol, lipophagy is activated in hepatocytes, serving as a defensive mechanism against injury to steatosis (Yan et al., 2019). However, it is impaired by chronic alcohol exposure, which is likely due to the activation of mTOR signaling and decreased lysosomal biogenesis in hepatocytes (Kounakis et al., 2019; Yang L. et al., 2019). There are growing number of herbs and natural products have been found to protect liver from injury induced by alcohol by mechanism of lipophagy stimulation. Corosolic acid, a compound derived from the leaves of *Langertroemia speciosa* L Pers., protected the liver from alcoholic-induced liver injury partially *via* restoring hepatic lipophagy due to mTORC1 suppression after AMPK activation (Guo et al., 2016). Another natural compound, quercitin, which is extensively found in many fruits and herbal plants, remarkably reversed the alcohol-induced blockade of TFEB nuclear localization, *via* restoring lysosome function and autophagic flux in livers of ethanol-fed C57BL6 mice (Li et al., 2019). Salvianolic acid A, a phenolic carboxylic acid extracted from *Salvia miltiorrhiza Bunge*, reduced hepatic steatosis induced by alcohol administration in rats. The action mechanism is attributed to enhanced autophagosome-lysosome fusion after restoring lysosomal cathepsin activities (Shi et al., 2018).

As a matter of fact, the field of lipophagy in liver diseases has yet to be fully developed. Its pathological role in different stages and circumstances of various liver disorders still needs to be revealed. Nevertheless, current studies concerning lipophagy have already provided new insights on lipid metabolism and energy homeostasis in the liver. It represents a promising path forward to the therapeutic of hepatic steatosis. Pharmaceutic agents including herbs, natural products or compounds targeting lipophagy in the liver deserve to be further investigated in future basic and clinic researches.

### Enhancing Fatty Acid β-Oxidation

Fatty acid could be oxidized by β-oxidation, α-oxidation, omega-oxidation, and peroxisomal oxidation, among which β-oxidation is the major type occurring in the mitochondria matrix (Wanders et al., 2015). In β-oxidation, two carbon subunits from fatty acids are removed repeatedly until the fatty acid carbon chain is fully degraded to form acetyl-CoA, which is further oxidized to carbon dioxide and H_2_O in the tricarboxylic acid cycle (TCA) (Canbay et al., 2007). β-oxidation plays a vital role in hepatic lipid consumption. A variety of proteins and enzymes are involved in the process of mitochondrial fatty acid β-oxidation, such as plasma membrane fatty acid binding protein (FABPpm) (Furuhashi and Hotamisligil, 2008), fatty acid transport protein (FATP) (Ouali et al., 2000), carnitine acylcarnitine translocase (CACT) (Pierre et al., 2007), carnitine palmitoyltransferases 1 and 2 (CPT1/2), etc. (Bonnefont et al., 2004; Houten and Wanders, 2010). More importantly, mitochondrial fatty acid β-oxidation is regulated by both transcriptional and posttranscriptional mechanisms. Peroxisome proliferator-activated receptors (PPARs) are activated by fatty acids, having specific roles in physiology of different tissues (Yu et al., 2003; Lamichane et al., 2018). In liver, PPARα controls many genes involved in mitochondrial fatty acid β-oxidation (Lamichane et al., 2018). In terms of posttranscriptional mechanism, the inhibition of CPT1 by malonyl-CoA is a vital regulatory step. The levels of malonyl-CoA in hepatocytes are regulated *via* degradation induced by malonyl-CoA decarboxylase and *via* production by acetyl-CoA carboxylase (ACC)(Park et al., 2002). PPARs-mediated activation persuades transcription of malonyl-CoA decarboxylase, and phosphorylated AMPK inactivated ACC (Saha and Ruderman, 2003). They stimulate mitochondrial fatty acid β-oxidation by reducing malonyl-CoA levels. Additionally, peroxisome proliferator activated receptor gamma coactivator 1-alpha (PGC-1α) has also been regarded as a factor of posttranscriptional regulation of β-oxidation (Fernandez-Marcos and Auwerx, 2011). The activation of PGC-1α is mediated by AMPK *via* SIRT1-mediated deacetylation (Canto and Auwerx, 2009).

Many herbs and active compounds protect liver from steatosis *via* regulation of fatty acid β-oxidation. Herbacetin is a dietary flavonoid with plenty of pharmacological activities. Its anti-hyperglycemic and anti-hyperlipidemic properties was associated with up-regulation of CPT to enhanced β-oxidation and hepatic lipid metabolism (Veeramani et al., 2018). Acteoside, a major compound isolated from leaves of *Aloysia citriodora* Palau (syn. *Lippia triphylla*), promoted lipolysis and fatty acid oxidation by enhancing mRNA expression level of adipose triglyceride lipase (ATGL) and CPT-1, and thus improved hepatic lipid metabolism (Zhang Y. et al., 2019). Cordycepin enhanced β-oxidation and suppressed lipid accumulation *via* regulating AMPK pathway and mitochondrial fusion in hepatocytes (Uen et al., 2018).

In China, the modified Longdan Xiegan Tang (mLXT, composed of Scutellaria baicalnsis Geprgi, Gardenia jasminoides, Adenophora capillaris, Akebia quinate, Plantago asiatica, Angelica sinensis, Rehmannia glutinosa, Alisma plantago-aquatica, Bupleurum gibraltaicum, and Glycyrrhiza uralensis) has been used clinically for various liver diseases such as NAFLD. It was found to activate hepatic expression of PPAR α and its target genes associated with fatty acid β-oxidation (Ren et al., 2019). Babaodan, a TCM, up-regulated the expression of CPT-1 and PPAR α in liver of HFD-fed mice with NAFLD, leading to the enhanced β-oxidation (Sheng et al., 2019). Rosa rugosa Thunb., another TCM, is used for treatment of cardiovascular diseases and diabetes, hypertension, hyperlipidemia, and inflammation. R. rugosa flavonoids, the major components in R. rugosa Thunb., were observed to up-regulate the mRNA expression of PPAR α and its downstream gene of acyl-coenzyme A oxidas X (ACOX) in a mouse model of hypertriglyceridemia (Baiyisaiti et al., 2019). Thereby, R. rugosa flavonoids could reduce TG in hepatocytes via rising β-oxidation. *Gynura procumbens* Merr., one of precious medicinal herbs of Asterceaes, up-regulated the mRNA expression of genes involved in β-oxidation, including PPAR α, CPT1 α, ACOX, fatty acid-binding proteins 5 (FABP5), stearoyl-coenzyme A desaturase-1 (SCD-1), glycerol-3-phosphate acyltransferase (mGPAT), microsomal triglyceride transfer protein (MTTP), to increase β-oxidation and efflux of fatty acids in liver of mice fed with MCD diet, and consequently decreased hepatic lipid accumulation (Liu Y. Y. et al., 2019). An herbal formula Gyeongshingangjeehwan 18 (GGEx18), composed of Laminaria japonica Aresch (Laminariaceae), Rheum palmatum L. (Polygonaceae) and Ephedra sinica Stapf (Ephedraceae), has traditionally been described to against obesity and related metabolic disease such as dyslipidemia. In HFD-fed mice receiving GGEx18, genes related to hepatic fatty acid β-oxidation was higher compared to mice fed with only HFD (Lim et al., 2018).

Evidence from recent studies has also indicated that some natural compounds promoted fatty acid oxidation by regulating the AMPK/PGC-1α signaling pathway. Yellow pigments, monascin, and ankaflavin, as secondary metabolites derived from monascus-fermented products, could reduce fatty acid accumulation partly mediated by the AMPK signaling activation and enhancement of β-oxidation by PGC-1α (Hsu et al., 2014). Myricetin, a natural flavonol with many biological activities, decreased PGC-1α acetylation through SIRT1 activation, and thus enhanced mitochondrial activity, suggesting its potential role in regulating hepatic lipid metabolism (Jung et al., 2017).

## Clinical Trials

Given to the encouraging effects of herbal medicines on liver diseases, plenty of clinical trials have been extensively performed. The potential therapeutic benefits of herbal medicines in patients with NAFLD have been reviewed in several papers in recent years (Xiao et al., 2013; Bedi et al., 2016; Perumpail et al., 2018). In present review, we focused on the efficacy of herbal medicines to mediate lipid metabolism and attenuate hepatic steatosis.

Dava Al-Balgham, as one of the traditional medicine products composed of *Nigella sativa* L., *Pistacia lentiscus* L., *Zataria multiflora* Boiss. (ZM), and *Trachyspermum ammi*, was tested for its effect on NAFLD by a randomized, double-blinded, placebo-controlled trial with 76 NAFLD patients. Placebo or Dava Al-Balgham were consumed with each meal for three months. The results showed that Dava Al-Balgham could cause weight loss and have anti-hypolipidemic effect (Hormati et al., 2019).

The effect of *Z. multiflora* supplementation on NAFLD was studied by a randomized double-blind placebo-controlled clinical trial. Total 85 patients with NAFLD were treated with ZM powder (700 mg) or placebo twice daily for 3 months. However, no significant difference between two ZM-treated groups and placebo groups regarding ALT, TNF-α, grade of fatty liver in ultrasonography, lipid profiles, and high sensitive C-reactive protein (hs-CRP), while it could improve insulin resistance in patients with NAFLD. Further studies with larger sample size and longer duration are recommended (Zamani et al., 2018).

A 12-weeks randomized, controlled, double-blind trial included with was 44 NAFLD patients, was performed to evaluate the efficacy of *Capparis spinosa* L. on disease regression of NAFLD. Patients are randomly divided into control (n=22) or caper (n=22) group. The caper group was treated with 40-50 g caper fruit pickles with meals every day. Results obtained after treatment of 12 weeks indicated that the grade of fatty liver and serum lipoproteins were improved by *C. spinosa* administration (Khavasi et al., 2017).

We further checked the registered clinical trials about testing effects of the herbs and natural products on fatty liver *via* the website of www.clinialtrial.gov. The intensively studied herbs and derived compounds are resveratrol, ginseng, and ginger, which were discussed in detail in following. Other herbs and some natural products that are undergoing or were performed clinical trials on fatty liver diseases are listed in **Table 3**.

**Table 3:** Registered clinical trials of herbs and natural products on fatty liver diseases (Referred to http://www.ClinicalTrials.gov website).

NCT number	Status	Conditions	Interventions	Outcome Measures	Population	Dates
NCT02030977	Completed	NAFLD	Resveratrol	ALT	Enrollment: 50 Age: 18 Years to 80 Years (Adult, Older Adult) Sex: All	Study Start: June 2012 Study Completion: March 2013
NCT01464801	Completed	Fatty liver	Resveratrol	Change in hepatic steatosis and inflammation Assessment of tolerability and side-effects	Enrollment: 28 Age: 18 Years to 70 Years (Adult, Older Adult) Sex: All	Study Start: September 2011 Study Completion: June 2015
NCT01446276	Completed	Obesity NAFLD	Resveratrol	Hepatic VLDL-TG secretion and peripheral VLDL-TG clearance Basal and insulin stimulated free fatty acid (FFA) and glucose turnover VLDL-TG oxidation Body composition (fat mass, fat-free mass, percent fat, visceral fat mass) lipoprotein lipase activity and fat cell size in abdominal and femoral adipose tissue biopsy Baseline data	Enrollment: 26 Age: 25 Years to 65 Years (Adult, Older Adult) Sex: Male	Study Start: November 2011 Study Completion: April 2014
NCT04130321	Not yet recruiting	Overweight Microtia Endotoxemia Metabolic Syndrome Insulin Resistance NAFLD	Camu camu (Myrciaria dubia)	Change in Gut Microbiota Composition and Diversity Change in fat accumulation in the liver Change in Endotoxemia Change in Intestinal permeability Change in Inflammation state of the tissue Change in Short chain and branched chain fatty acids in the feces Change in gut health Change in stool consistency Change in Glucose homeostasis Change in Lipid profile and 8 more	Enrollment: 32 Age: 18 Years to 75 Years (Adult, Older Adult) Sex: All	Study Start: January 6, 2020 Study Completion: June 30, 2022
NCT0394512	Completed	Liver Dysfunction	Red ginseng	Liver enzyme	Enrollment: 94 Age: 37 Years to 63 Years (Adult) Sex: All	Study Start: January 1, 2018 Study Completion: December 31, 2018
NCT03260543	Completed	NAFLD	Fermented ginseng powder	Changes of ALT Changes of Liver function index Changes of fatty liver grade Changes of lipid metabolism index Changes of total antioxidant capacity Changes of imflammation index Changes of Multidimensional Fatigue Scale	Enrollment: 90 Age: 19 Years to 70 Years (Adult, Older Adult) Sex: All	Study Start: July 2016 Study Completion: August 2017
NCT04049396	Completed	NAFLD	Berberine	ALT; AST; ALP; fasting blood sugar; total cholesterol; LDL-Cholesterol; HDL - Cholesterol; TG	Enrollment: 50 Age: 18 Years to 65 Years (Adult, Older Adult) Sex: All	Study Start: October 1, 2018 Study Completion: June 15, 2019
NCT02535195	Completed	NAFLD	Ginger	Serum levels of the ALT liver enzyme Serum levels of the AST liver enzyme controlled attenuation parameter(CAP) score	Enrollment: 60 Age: 18 Years to 70 Years (Adult, Older Adult) Sex: All	Study Start: March 2013 Study Completion: August 2015
NCT02289235	Enrolling by invitation	Fatty Liver Diabetes Mellitus, Type 2	Ginger	Change in ALT level Change in AST level Change in score of fatty liver in fibroscan Change in Gama GT (#- glutamyl transpeptidase) levels Number of patients with adverse events	Enrollment: 90 Age: 20 Years to 65 Years (Adult, Older Adult) Sex: All	Study Start: November 1, 2018 Study Completion: December 1, 2019
NCT03864783	Recruiting	NAFLD Insulin Resistance Glucose Tolerance Impaired Obesity, Abdominal	Curcumin (Meriva^®^)	Curcumin’s effect on steatosis Total amino acids in plasma Total amino acids in plasma Curcumin’s effect on plasma concentration of urea Curcumin’s effect on urin concentration of urea Curcumin’s effect on serum concentration of inflammatory marker interleukin (IL)-1b Curcumin’s effect on serum concentration of inflammatory marker IL-2 Curcumin’s effect on serum concentration of inflammatory marker IL-6 Curcumin’s effect on serum concentration of inflammatory marker IL-10 Curcumin’s effect on serumconcentration of inflammatory marker tumor necrosis factor (TNF)- alpha Curcumin’s effect on plasma concentration of adipokines and 34 more	Enrollment: 40 Age: 20 Years and older (Adult, Older Adult) Sex: Male	Study Start: March 5, 2019 Study Completion: October 2020
NCT03073343	Recruiting	Non-Alcoholic Fatty Liver Disease Non Insulin Dependent Diabetes ALT	Betaine	ALT	Enrollment: 48 Age: 18 Years to 75 Years (Adult, Older Adult) Sex: All	Study Start: November 12, 2013 Study Completion: June 30, 2020
NCT02973295	Recruiting	NAFLD	Silymarin	Change (Reduction) of parameters of liver steatosis defined by CAP (Controlled Attenuation Parameter) and liver fibrosis defined by LSM (liver stiffness measurements) during the 6 months period Change in liver enzymes in period of 6 months Change in insulin resistance in period of 6 months Change in lipidogram in period of 6 months	Enrollment: 400 Age: 18 Years to 70 Years (Adult, Older Adult) Sex: All	Study Start: September 20, 2019 Study Completion: June 30, 2021
NCT02929901	Completed	Type 2 Diabetes Nonalcoholic Fatty Liver	Caffeine and chlorogenic acid	Hepatic steatosis Glucose Glycated hemoglobin (HBA1C) ALT AST hs- CRP) gut microbiota	Enrollment: 200 Age: 30 Years to 65 Years (Adult, Older Adult) Sex: All	Study Start: December 2016 Study Completion: March 2019
NCT02908152	Unknown status	Type 2 Diabetes Nonalcoholic Fatty Liver	Curcumin	Hepatic steatosis Glucose HBA1C ALT AST	Enrollment: 50 Age: 30 Years to 65 Years (Adult, Older Adult) Sex: All	Study Start: February 2017 Study Completion: October 2017
NCT02006498	Completed	NAFLD	Sillymarin	To assess the efficacy of Silymarin as defined by an improvement in non-alcoholic steatosis (NAS) activity score by at least 30% from baseline compared to placebo To assess the safety and adverse event profile of Silymarin compared to placebo	Enrollment: 99 Age: 18 Years and older (Adult, Older Adult) Sex: All	Study Start: June 2012 Study Completion: December 2015
NCT01940263	Completed	NAFLD	Anthocyanin	Biomarkers related to oxidative stress Biomarkers related to inflammation	Enrollment: 63 Age: 18 Years to 65 Years (Adult, Older Adult) Sex: All	Study Start: June 2013 Study Completion: June 2014
NCT02307344	Unknown status	Nonalcoholic Steatohepatitis Liver Steatosis	Nigella sativa L.	Effect of Nigella Sativa on Liver Triglyceride Concentration Effect of Nigella Sativa on Improvement in NASH Activity Index Effect of Nigella Sativa on Fibrosis Staging	Enrollment: 100 Age: 18 Years and older (Adult, Older Adult) Sex: All	Study Start: January 2015 Study Completion: January 2017
NCT02303314	Completed	NAFLD	Trigonella Foenum-graecum Seed Extract	Liver stiffness change	Enrollment: 35 Age: 18 Years to 70 Years (Adult, Older Adult) Sex: All	Study Start: November 2014 Study Completion: September 2017
NCT01707914	Completed	NAFLD	Chinese bayberry juice (Myrica rubra)	Plasma lipids profile	Enrollment: 44 Age: 18 Years to 25 Years (Adult) Sex: All	Study Start: June 2012
NCT01677325	Completed	NAFLD	Drug: Chinese herb (YiQiSanJu) (Angelica sinensis, Rehmannia, Cinnamomum cassia, Glycyrrhiza uralensis, Eucommia ulmoides, Achyranthes bidentate, Lycium chinense)	The CT ratio of liver/spleen BMI(Body Mass Index) liver function lipid profile NEFA HOMA index adiponectin IL-6 hs-CRP (C-reactive protein) TNF-a leptin	Enrollment: 40 Age: 18 Years to 65 Years (Adult, Older Adult) Sex: Male	Study Start: January 2007 Study Completion: January 2008
NCT01210989	Completed	NAFLD	Phyllanthus urinaria L.	Histologic NAFLD activity score ALT normalization Metabolic endpoints Changes in magnetic resonance spectroscopy Liver stiffness measurement Biomarkers of NASH and liver fibrosis	Enrollment: 60 Age: 18 Years to 70 Years (Adult, Older Adult) Sex: All	Study Start: May 2010 Study Completion: May 2012
NCT00816465	Completed	NAFLD	Hoodia gordonii (Masson) Sweet ex Decne.	Decreased insulin resistance Safety Reduced hepatic injury Reduced weight/BMI/abdominal circumference	Enrollment: 20 Age: 18 Years to 65 Years (Adult, Older Adult) Sex: All	Study Start: May 2009 Study Completion: August 2010

Resveratrol is a stilbenoid and a phytoalexin generated by several plants, such as red grapes in response to stimuli (Hasan and Bae, 2017). It is an activator of AMPK and SIRT1, and thus has a critical role in promoting fat breakdown and removal from the liver, preventing liver damage and inhibiting the progression of NAFLD (Shang et al., 2008; Charytoniuk et al., 2017; Theodotou et al., 2019). Resveratrol has been involved in three trials (NCT01446276; NCT01464801; NCT02030977) included patients of fatty liver, NAFLD, and obesity.

Another herb, ginseng, has been traditionally used for more than 2,000 years with various biological effects. A great deal of preclinical studies have demonstrated the protective effects of ginseng on liver diseases, including ALD and NAFLD. Korean Red Ginseng (Panax ginseng) (Park et al., 2017) enhanced the decreased phosphorylation of AMPK induced by ethanol consumption. Notably, it reduced the accumulation of fat in hepatocytes caused by ethanol *via* regulation of SREBP-1, SIRT-1 and PPAR-α (Huu Tung et al., 2012; Park et al., 2017). Clinical trial (NCT0394512) has been performed to study the effect of red ginseng on liver dysfunction. Fermented ginseng powder has also been tested to study its efficacy on NAFLD (NCT03260543).

Ginger is the root of *Zingiber officinale* Roscoe and is one of the most used spices in many countries (Huu Tung et al., 2012). It contains active compounds, such as shogaol, gingerol, zingerone, and β-bisabolene. It has been shown that ginger can reduce insulin resistance and serum TG level in patients with Type II diabetes and hyperlipidemia (Arablou et al., 2014). In a randomized, double-blind, placebo-controlled clinical trial with 44 patients of NAFLD, ginger supplementation significantly reduced the levels of ALT, inflammatory cytokines, γ-glutamyl transferase, as well as hepatic steatosis grade and the insulin resistance index in comparison to the control group. Another clinical trial of ginger supplement on fatty liver or Type 2 Diabetes Mellitus is still undergoing (NCT02289235).

## Meta-Analysis Studies

HuoXueHuaYu (HXHY), a TCM formula, has been widely used in clinic for patients with NAFLD. Cai et al. performed a meta-analysis of randomized controlled trial of HXHY in NAFLD. There are 13 studies involving 1429 patients which 654 patients receiving conventional treatment group and 775 patients belonged to HXHY group. HXHY showed better ability on lowing TC and TG levels than that of conversational treatment. HXHY might be an effective and safe therapy for NAFLD, and trials with rigorous design, multicenter, large-scale, and high-quality worldwide are still expected (Cai et al., 2019).

Erchen Decoction (ECD), a TCM formula, is often used in the therapy of various diseases. A meta-analysis of the efficacy of ECD for the treatment of NAFLD by PRISMA systematic review standard has been performed. Seven randomized controlled trial with a total of 1951 participants were included in this study. The analysis results showed that patients with ECD treatment showed an improved status compared to the conventional treatment. Longer follow-up periods and larger-scale randomized controlled trial are still required to evaluate the efficacy of ECD in NAFLD (Li et al., 2017).

The efficiency and safety of a famous TCM Danshen in the treatment of NAFLD has also been analyzed by a meta-analysis study. Eight randomized controlled trials with 800 patients of NAFLD were identified. The results indicated that Danshen had improved total effectiveness rate, lower level of TC, TG, LDL, ALT, and AST, suggesting that Danshen may have potential effects on NAFLD, while multicenter large-sample randomized clinical trials are still expected to confirm the efficacy and safety of Danshen (Peng et al., 2016).

Another study performed by Narjes et al. on 2017 has evaluated the efficiency of all kinds of TCM on the treatment of NAFLD. Literature were searched on China National Knowledge and PubMed from 1995 to 2010. Total 5904 patients from 62 randomized controlled trials were included for meta-analysis. Results showed that TCM had a better effect on the normalization of ALT level and disappearance of radiological steatosis for the patients of NAFLD. Finally, authors concluded that TCM is of modest benefit to the therapy of NAFLD (Shi et al., 2012).

## Conclusions and Perspectives

Due to the positive efficacy and minimal side effects, herbal medicines have obtained increasing attention as alternative therapeutic agents for liver disorders and dyslipidemia. Increasing evidence from laboratory studies suggests that many herbs, natural products, and derived compounds could inhibit the progression of hepatic steatosis. A variety of mechanisms have been demonstrated to be implicated in preventing hepatic steatosis and modulating lipid metabolism by herbs, including reducing hepatocyte fatty acid uptake and trafficking, reducing hepatic *de novo* lipogenesis, increasing lipolysis, inducing lipophagy, enhancing fatty acid β-oxidation. In particular, SREBP-1c, PPARα, AMPK, and SIRT1 signaling pathways have been highlighted as crucial molecular targets of action mechanisms by which herbal medicines regulate hepatic lipid metabolism. Current clinical evidences and meta-analysis showing the positive impacts of herbal medicines on the hepatic lipid metabolism pathways are still not strong enough. Further multicenter large-sample randomized clinical trials are still required to confirm the efficacy and safety of herbal medicines on hepatic lipid metabolism. Herbs mix and single medical plants as well as their components have been widely applied in the treatment of NAFLD. We consider the main actor should be the active components. For both herbs mix and single medical plants, they are containing many compounds, which may act synergistically in ways to enhance the therapeutic effects. Identifying the active components in herbs is a crucial and significant subject for the development of TCM. Currently, network pharmacology-based strategy has been extensively used for the prediction of the active components from herbs. Network pharmacology is an approach based on systems biology, poly-pharmacology, and molecular networks, to analyze relationships between drugs and diseases in recent decade, which has attracted considerable attention among Chinese medicine researchers for its ability in predicting and illustrating interactive relationships between numerous components and targets of herbal medicines. Network-based pharmacological analysis is a desirable approach as well as a good tool of in silico prediction for investigating the mechanisms of action for herbs and formulae and their potential bioactive components at molecular and systematic levels, which renders more effective subsequent exploration with experimental approaches. With the promising and effective prediction, subsequently validation experiments in laboratory and bench would be performed to confirm their pivotal role. In conclusion, herbal medicines have the potency to be alternative and complementary medical therapies to current pharmaceuticals for the treatment of liver diseases with lipid metabolism disorder.

## Author Contributions

YF designed and conceived the study. SL and YF retrieved and analyzed the data, and drafted the manuscript. SL, YX, WG, FC, CZ, HT, and NW discussed and revised the manuscript. All authors confirmed final version of the manuscript.

## Funding

This research was partially supported by the Research Council of the University of Hong Kong (project codes: 104004092 and 104004460), Wong’s donation (project code: 200006276), a donation from the Gaia Family Trust of New Zealand (project code: 200007008), the Research Grants Committee (RGC) of Hong Kong, HKSAR (project codes: 740608, 766211, 17152116, and 17121419), and Health and Medical Research Fund (project codes: 15162961, 16171511, and 16172751).

## Conflict of Interest

The authors declare that the research was conducted in the absence of any commercial or financial relationships that could be construed as a potential conflict of interest.

## References

[B1] Ahn S. H. Lee K. P. Kim K. Choi J. Y. Park S. Y. Cheon J. H. (2019). Dansameum regulates hepatic lipogenesis and inflammation *in vitro* and *in vivo*. Food Sci. Biotechnol. 28 (5), 1543–1551. 10.1007/s10068-019-00579-8 31695954 PMC6811489

[B2] Albhaisi S. Sanyal A. (2018). Recent advances in understanding and managing non-alcoholic fatty liver disease. F1000Res 7. 10.12688/f1000research.14421.1 PMC5998003 29946426

[B3] Al-Dayyat H. M. Rayyan Y. M. Tayyem R. F. (2018). Non-alcoholic fatty liver disease and associated dietary and lifestyle risk factors. Diabetes Metab. Syndr. 12 (4), 569–575. 10.1016/j.dsx.2018.03.016 29571977

[B4] Ansari A. Bose S. Patra J. K. Shin N. R. Lim D. W. Kim K. W. (2018). A Controlled Fermented Samjunghwan Herbal Formula Ameliorates Non-alcoholic Hepatosteatosis in HepG2 Cells and OLETF Rats. Front. Pharmacol. 9, 596. 10.3389/fphar.2018.00596 29971000 PMC6018163

[B5] Arablou T. Aryaeian N. Valizadeh M. Sharifi F. Hosseini A. Djalali M. (2014). The effect of ginger consumption on glycemic status, lipid profile and some inflammatory markers in patients with type 2 diabetes mellitus. Int. J. Food Sci. Nutr. 65 (4), 515–520. 10.3109/09637486.2014.880671 24490949

[B6] Aronow W. S. (2006). Management of hyperlipidemia with statins in the older patient. Clin. Interv. Aging 1 (4), 433–438. 10.2147/ciia.2006.1.4.433 18046920 PMC2699649

[B7] Ayuob N. N. Abdel-Hamid A. Helal G. M. M. Mubarak W. A. (2019). Thymoquinone reverses nonalcoholic fatty liver disease (NAFLD) associated with experimental hypothyroidism. Rom. J. Morphol. Embryol. 60 (2), 479–486. 31658321

[B8] Baiyisaiti A. Wang Y. Zhang X. Chen W. Qi R. (2019). Rosa rugosa flavonoids exhibited PPARalpha agonist-like effects on genetic severe hypertriglyceridemia of mice. J. Ethnopharmacol. 240, 111952. 10.1016/j.jep.2019.111952 31100436

[B9] Bedi O. Bijjem K. R. V. Kumar P. Gauttam V. (2016). Herbal Induced Hepatoprotection and Hepatotoxicity: A Critical Review. Indian J. Physiol. Pharmacol. 60 (1), 6–21. 29953177

[B10] Bonnefont J. P. Djouadi F. Prip-Buus C. Gobin S. Munnich A. Bastin J. (2004). Carnitine palmitoyltransferases 1 and 2: biochemical, molecular and medical aspects. Mol. Aspects Med. 25 (5-6), 495–520. 10.1016/j.mam.2004.06.004 15363638

[B11] Cai Y. Liang Q. Chen W. Chen M. Chen R. Zhang Y. (2019). Evaluation of HuoXueHuaYu therapy for nonalcoholic fatty liver disease: a systematic review and meta-analysis of randomized controlled trial. BMC Complement Altern. Med. 19 (1), 178. 10.1186/s12906-019-2596-3 31324247 PMC6642602

[B12] Canbay A. Bechmann L. Gerken G. (2007). Lipid metabolism in the liver. Z. Gastroenterol. 45 (1), 35–41. 10.1055/s-2006-927368 17236119

[B13] Canto C. Auwerx J. (2009). PGC-1alpha, SIRT1 and AMPK, an energy sensing network that controls energy expenditure. Curr. Opin. Lipidol. 20 (2), 98–105. 10.1097/MOL.0b013e328328d0a4 19276888 PMC3627054

[B14] Cao S. Zhou Y. Xu P. Wang Y. Yan J. Bin W. (2013). Berberine metabolites exhibit triglyceride-lowering effects via activation of AMP-activated protein kinase in Hep G2 cells. J. Ethnopharmacol. 149 (2), 576–582. 10.1016/j.jep.2013.07.025 23899453

[B15] Cao H. Tuo L. Tuo Y. Xia Z. Fu R. Liu Y. (2017). Immune and Metabolic Regulation Mechanism of Dangguiliuhuang Decoction against Insulin Resistance and Hepatic Steatosis. Front. Pharmacol. 8, 445. 10.3389/fphar.2017.00445 28736524 PMC5500616

[B16] Cao H. Li S. Xie R. Xu N. Qian Y. Chen H. (2018). Exploring the Mechanism of Dangguiliuhuang Decoction Against Hepatic Fibrosis by Network Pharmacology and Experimental Validation. Front. Pharmacol. 9, 187. 10.3389/fphar.2018.00187 29556199 PMC5844928

[B17] Chao H. W. Chao S. W. Lin H. Ku H. C. Cheng C. F. (2019). Homeostasis of Glucose and Lipid in Non-Alcoholic Fatty Liver Disease. Int. J. Mol. Sci. 20 (2). 10.3390/ijms20020298 PMC6359196 30642126

[B18] Charytoniuk T. Drygalski K. Konstantynowicz-Nowicka K. Berk K. Chabowski A. (2017). Alternative treatment methods attenuate the development of NAFLD: A review of resveratrol molecular mechanisms and clinical trials. Nutrition 34, 108–117. 10.1016/j.nut.2016.09.001 28063505

[B19] Chatterjee C. Sparks D. L. (2011). Hepatic lipase, high density lipoproteins, and hypertriglyceridemia. Am. J. Pathol. 178 (4), 1429–1433. 10.1016/j.ajpath.2010.12.050 21406176 PMC3078429

[B20] Choi J. Y. Kwon E. Y. Choi M. S. (2019). Elucidation of the Metabolic and Transcriptional Responses of an Oriental Herbal Medicine, Bangpungtongseong-san, to Nonalcoholic Fatty Liver Disease in Diet-Induced Obese Mice. J. Med. Food 22 (9), 928–936. 10.1089/jmf.2018.4383 31390281

[B21] Cui Y. Chang R. Zhang T. Zhou X. Wang Q. Gao H. (2019). Chinese Herbal Formula (CHF03) Attenuates Non-Alcoholic Fatty Liver Disease (NAFLD) Through Inhibiting Lipogenesis and Anti-Oxidation Mechanisms. Front. Pharmacol. 10, 1190. 10.3389/fphar.2019.01190 31680967 PMC6803500

[B22] Czaja M. J. (2016). Function of Autophagy in Nonalcoholic Fatty Liver Disease. Dig. Dis. Sci. 61 (5), 1304–1313. 10.1007/s10620-015-4025-x 26725058 PMC4838507

[B23] Dang Y. Hao S. Zhou W. Zhang L. Ji G. (2019). The traditional Chinese formulae Ling-gui-zhu-gan decoction alleviated non-alcoholic fatty liver disease via inhibiting PPP1R3C mediated molecules. BMC Complement Altern. Med. 19 (1), 8. 10.1186/s12906-018-2424-1 30616587 PMC6323852

[B24] Dong H. Lu F. E. Zhao L. (2012). Chinese herbal medicine in the treatment of nonalcoholic fatty liver disease. Chin. J. Integr. Med. 18 (2), 152–160. 10.1007/s11655-012-0993-2 22311412

[B25] Eissing L. Scherer T. Todter K. Knippschild U. Greve J. W. Buurman W. A. (2013). De novo lipogenesis in human fat and liver is linked to ChREBP-beta and metabolic health. Nat. Commun. 4, 1528. 10.1038/ncomms2537 23443556 PMC3740744

[B26] Feng Q. Liu W. Baker S. S. Li H. Chen C. Liu Q. (2017). Multi-targeting therapeutic mechanisms of the Chinese herbal medicine QHD in the treatment of non-alcoholic fatty liver disease. Oncotarget 8 (17), 27820–27838. 10.18632/oncotarget.15482 28416740 PMC5438611

[B27] Feng W. W. Kuang S. Y. Tu C. Ma Z. J. Pang J. Y. Wang Y. H. (2018). Natural products berberine and curcumin exhibited better ameliorative effects on rats with non-alcohol fatty liver disease than lovastatin. BioMed. Pharmacother. 99, 325–333. 10.1016/j.biopha.2018.01.071 29353208

[B28] Fernandez-Marcos P. J. Auwerx J. (2011). Regulation of PGC-1alpha, a nodal regulator of mitochondrial biogenesis. Am. J. Clin. Nutr. 93 (4), 884S–8890. 21289221 10.3945/ajcn.110.001917 PMC3057551

[B29] Furuhashi M. Hotamisligil G. S. (2008). Fatty acid-binding proteins: role in metabolic diseases and potential as drug targets. Nat. Rev. Drug Discovery 7 (6), 489–503. 10.1038/nrd2589 18511927 PMC2821027

[B30] Guo X. Cui R. Zhao J. Mo R. Peng L. Yan M. (2016). Corosolic acid protects hepatocytes against ethanol-induced damage by modulating mitogen-activated protein kinases and activating autophagy. Eur. J. Pharmacol. 791, 578–588. 10.1016/j.ejphar.2016.09.031 27663281

[B31] Guo Y. Li J. X. Mao T. Y. Zhao W. H. Liu L. J. Wang Y. L. (2017). Targeting Sirt1 in a rat model of high-fat diet-induced non-alcoholic fatty liver disease: Comparison of Gegen Qinlian decoction and resveratrol. Exp. Ther. Med. 14 (5), 4279–4287. 10.3892/etm.2017.5076 29104641 PMC5658732

[B32] Hasan M. Bae H. (2017). “An Overview of Stress-Induced Resveratrol Synthesis in Grapes: Perspectives for Resveratrol-Enriched Grape Products. Molecules 22 (2). 10.3390/molecules22020294 PMC6155908 28216605

[B33] Heeboll S. Vilstrup H. Gronbaek H. (2018). “[Treatment of non-alcoholic fatty liver disease]. Ugeskr Laeger 180 (31). 30064623

[B34] Ho C. Gao Y. Zheng D. Liu Y. Shan S. Fang B. (2019). Alisol A attenuates high-fat-diet-induced obesity and metabolic disorders via the AMPK/ACC/SREBP-1c pathway. J. Cell Mol. Med. 23 (8), 5108–5118. 10.1111/jcmm.14380 31144451 PMC6653754

[B35] Hormati A. Tooiserkany F. Mohammadbeigi A. Aliasl F. Dehnavi H. M. (2019). Effect of an Herbal Product on the Serum Level of Liver Enzymes in Patients with Non-Alcoholic Fatty Liver Disease: A Randomized, Double-Blinded, Placebo-Controlled Trial. Iranian Red Crescent Med. J. 21 (7), 7. 10.5812/ircmj.91024

[B36] Houten S. M. Wanders R. J. (2010). A general introduction to the biochemistry of mitochondrial fatty acid beta-oxidation. J. Inherit. Metab. Dis. 33 (5), 469–477. 10.1007/s10545-010-9061-2 20195903 PMC2950079

[B37] Hsu W. H. Chen T. H. Lee B. H. Hsu Y. W. Pan T. M. (2014). Monascin and ankaflavin act as natural AMPK activators with PPARalpha agonist activity to down-regulate nonalcoholic steatohepatitis in high-fat diet-fed C57BL/6 mice. Food Chem. Toxicol. 64, 94–103. 10.1016/j.fct.2013.11.015 24275089

[B38] Huang L. Cheng Y. Huang K. Zhou Y. Ma Y. Zhang M. (2018). Ameliorative effect of Sedum sarmentosum Bunge extract on Tilapia fatty liver *via* the PPAR and P53 signaling pathway. Sci. Rep. 8 (1), 8456. 29855491 10.1038/s41598-018-26084-2 PMC5981579

[B39] Huang X. Chen W. Yan C. Yang R. Chen Q. Xu H. (2019). Gypenosides improve the intestinal microbiota of non-alcoholic fatty liver in mice and alleviate its progression. BioMed. Pharmacother. 118, 109258. 10.1016/j.biopha.2019.109258 31545283

[B40] Huu Tung N. Uto T. Morinaga O. Kim Y. H. Shoyama Y. (2012). Pharmacological effects of ginseng on liver functions and diseases: a minireview. Evid. Based Complement Alternat. Med. 2012, 173297. 22997528 10.1155/2012/173297 PMC3446728

[B41] Ibrahim S. H. Hirsova P. Malhi H. Gores G. J. (2016). Animal Models of Nonalcoholic Steatohepatitis: Eat, Delete, and Inflame. Dig. Dis. Sci. 61 (5), 1325–1336. 10.1007/s10620-015-3977-1 26626909 PMC4838538

[B42] Ipsen D. H. Lykkesfeldt J. Tveden-Nyborg P. (2018). Molecular mechanisms of hepatic lipid accumulation in non-alcoholic fatty liver disease. Cell Mol. Life Sci. 75 (18), 3313–3327. 10.1007/s00018-018-2860-6 29936596 PMC6105174

[B43] Issa D. Patel V. Sanyal A. J. (2018). Future therapy for non-alcoholic fatty liver disease. Liver Int. 38 Suppl 1, 56–63. 10.1111/liv.13676 29427492

[B44] Jang J. Jung Y. Chae S. Cho S. H. Yoon M. Yang H. (2018). Gangjihwan, a polyherbal composition, inhibits fat accumulation through the modulation of lipogenic transcription factors SREBP1C, PPARgamma and C/EBPalpha. J. Ethnopharmacol. 210, 10–22. 10.1016/j.jep.2017.08.024 28842339

[B45] Jung H. Y. Lee D. Ryu H. G. Choi B. H. Go Y. Lee N. (2017). Myricetin improves endurance capacity and mitochondrial density by activating SIRT1 and PGC-1alpha. Sci. Rep. 7 (1), 6237. 28740165 10.1038/s41598-017-05303-2 PMC5524912

[B46] Kang H. Koppula S. (2014). Houttuynia cordata attenuates lipid accumulation via activation of AMP-activated protein kinase signaling pathway in HepG2 cells. Am. J. Chin. Med. 42 (3), 651–664. 10.1142/S0192415X14500426 24871657

[B47] Kang O. H. Kim S. B. Mun S. H. Seo Y. S. Hwang H. C. Lee Y. M. (2015). Puerarin ameliorates hepatic steatosis by activating the PPARalpha and AMPK signaling pathways in hepatocytes. Int. J. Mol. Med. 35 (3), 803–809. 10.3892/ijmm.2015.2074 25605057

[B48] Kawano Y. Cohen D. E. (2013). Mechanisms of hepatic triglyceride accumulation in non-alcoholic fatty liver disease. J. Gastroenterol. 48 (4), 434–441. 10.1007/s00535-013-0758-5 23397118 PMC3633701

[B49] Khalil M. Khalifeh H. Baldini F. Salis A. Damonte G. Daher A. (2019). Antisteatotic and antioxidant activities of Thymbra spicata L. extracts in hepatic and endothelial cells as in vitro models of non-alcoholic fatty liver disease. J. Ethnopharmacol. 239, 111919. 31029756 10.1016/j.jep.2019.111919

[B50] Khavasi N. Somi M. H. Khadem E. Faramarzi E. Ayati M. H. Fazljou S. M. B. (2017). Effect of Daily Caper Fruit Pickle Consumption on Disease Regression in Patients with Non-Alcoholic Fatty Liver Disease: a Double-Blinded Randomized Clinical Trial. Adv. Pharm. Bull. 7 (4), 645–650. 10.15171/apb.2017.077 29399555 PMC5788220

[B51] Kounakis K. Chaniotakis M. Markaki M. Tavernarakis N. (2019). Emerging Roles of Lipophagy in Health and Disease. Front. Cell Dev. Biol. 7, 185. 10.3389/fcell.2019.00185 31552248 PMC6746960

[B52] Kumashiro N. Yoshimura T. Cantley J. L. Majumdar S. K. Guebre-Egziabher F. Kursawe R. (2013). Role of patatin-like phospholipase domain-containing 3 on lipid-induced hepatic steatosis and insulin resistance in rats. Hepatology 57 (5), 1763–1772. 10.1002/hep.26170 23175050 PMC3597437

[B53] Kwanten W. J. Martinet W. Michielsen P. P. Francque S. M. (2014). Role of autophagy in the pathophysiology of nonalcoholic fatty liver disease: a controversial issue. World J. Gastroenterol. 20 (23), 7325–7338. 10.3748/wjg.v20.i23.7325 24966603 PMC4064078

[B54] Lamichane S. Dahal Lamichane B. Kwon S. M. (2018). Pivotal Roles of Peroxisome Proliferator-Activated Receptors (PPARs) and Their Signal Cascade for Cellular and Whole-Body Energy Homeostasis. Int. J. Mol. Sci. 19 (4). 10.3390/ijms19040949 PMC5979443 29565812

[B55] Lass A. Zimmermann R. Oberer M. Zechner R. (2011). Lipolysis - a highly regulated multi-enzyme complex mediates the catabolism of cellular fat stores. Prog. Lipid Res. 50 (1), 14–27. 10.1016/j.plipres.2010.10.004 21087632 PMC3031774

[B56] Lee J. E. Kim E. J. Kim M. H. Hong J. Yang W. M. (2016). Polygonatum stenophyllum improves menopausal obesity via regulation of lipolysis-related enzymes. J. Nat. Med. 70 (4), 789–796. 10.1007/s11418-016-1018-9 27324797

[B57] Lee Y. H. Jin B. Lee S. H. Song M. Bae H. Min B. J. (2016). Herbal Formula HT048 Attenuates Diet-Induced Obesity by Improving Hepatic Lipid Metabolism and Insulin Resistance in Obese Rats”. Molecules 21 (11). 10.3390/molecules21111424 PMC6274173 27792149

[B58] Lee J. H. Baek S. Y. Jang E. J. Ku S. K. Kim K. M. Ki S. H. (2018). Oxyresveratrol ameliorates nonalcoholic fatty liver disease by regulating hepatic lipogenesis and fatty acid oxidation through liver kinase B1 and AMP-activated protein kinase. Chem. Biol. Interact. 289, 68–74. 10.1016/j.cbi.2018.04.023 29702089

[B59] Li X. Li Z. Xue M. Ou Z. Liu M. Yang M. (2013). Fructus Xanthii attenuates hepatic steatosis in rats fed on high-fat diet. PloS One 8 (4), e61499. 10.1371/journal.pone.0061499 23585904 PMC3621865

[B60] Li W. Li Y. Wang Q. Yang Y. (2014). Crude extracts from Lycium barbarum suppress SREBP-1c expression and prevent diet-induced fatty liver through AMPK activation. BioMed. Res. Int. 2014, 196198. 25013763 10.1155/2014/196198 PMC4071778

[B61] Li Y. Zhao J. Zheng H. Zhong X. Zhou J. Hong Z. (2014). Treatment of Nonalcoholic Fatty Liver Disease with Total Alkaloids in Rubus aleaefolius Poir through Regulation of Fat Metabolism. Evid. Based Complement Alternat. Med. 2014, 768540. 10.1155/2014/768540 25404949 PMC4212541

[B62] Li Z. Xu J. Zheng P. Xing L. Shen H. Yang L. (2015). Hawthorn leaf flavonoids alleviate nonalcoholic fatty liver disease by enhancing the adiponectin/AMPK pathway. Int. J. Clin. Exp. Med. 8 (10), 17295–17307. 26770322 PMC4694222

[B63] Li W. S. Wu Y. Ge W. Z. Fan L. Sun W. (2017). A herbal formula Erchen decoction for non-alcoholic fatty liver disease: a systematic review and meta-analysis of randomized controlled trials. Int. J. Clin. Exp. Med. 10 (6), 9110–9116.

[B64] Li C. H. Tang S. C. Wong C. H. Wang Y. Jiang J. D. Chen Y. (2018). Berberine induces miR-373 expression in hepatocytes to inactivate hepatic steatosis associated AKT-S6 kinase pathway. Eur. J. Pharmacol. 825, 107–118. 10.1016/j.ejphar.2018.02.035 29477657

[B65] Li Y. Y. Tang D. Du Y. L. Cao C. Y. Nie Y. Q. Cao J. (2018). Fatty liver mediated by peroxisome proliferator-activated receptor-alpha DNA methylation can be reversed by a methylation inhibitor and curcumin. J. Dig. Dis. 19 (7), 421–430. 10.1111/1751-2980.12610 29802754

[B66] Li Y. Y. Chen M. Wang J. Guo X. P. Xiao L. Liu P. Y. (2019). Quercetin ameliorates autophagy in alcohol liver disease associated with lysosome through mTOR-TFEB pathway. J. Funct. Foods 52, 177–185. 10.1016/j.jff.2018.10.033

[B67] Liang Z. E. Zhang Y. P. Tang K. R. Deng Y. J. Liang Y. Q. Liang S. (2019). Anti-inflammation effect via TLR4-mediated MyD88-dependent and -independent signalling pathways in non-alcoholic fatty liver disease rats: Chinese herb formula. Int. J. Clin. Exp. Med. 12 (3), 2265–2277.

[B68] Liao C. C. Ou T. T. Huang H. P. Wang C. J. (2014). The inhibition of oleic acid induced hepatic lipogenesis and the promotion of lipolysis by caffeic acid via up-regulation of AMP-activated kinase. J. Sci. Food Agric. 94 (6), 1154–1162. 10.1002/jsfa.6386 24027117

[B69] Lim J. Lee H. Ahn J. Kim J. Jang J. Park Y. (2018). The polyherbal drug GGEx18 from Laminaria japonica, Rheum palmatum, and Ephedra sinica inhibits hepatic steatosis and fibroinflammtion in high-fat diet-induced obese mice. J. Ethnopharmacol. 225, 31–41. 10.1016/j.jep.2018.06.034 29958960

[B70] Liu K. Czaja M. J. (2013). Regulation of lipid stores and metabolism by lipophagy. Cell Death Differ. 20 (1), 3–11. 10.1038/cdd.2012.63 22595754 PMC3524634

[B71] Liu L. Yang M. Lin X. Li Y. Liu C. Yang Y. (2013). Modulation of hepatic sterol regulatory element-binding protein-1c-mediated gene expression contributes to Salacia oblonga root-elicited improvement of fructose-induced fatty liver in rats. J. Ethnopharmacol. 150 (3), 1045–1052. 10.1016/j.jep.2013.10.020 24157375

[B72] Liu Z. L. Xie L. Z. Zhu J. Li G. Q. Grant S. J. Liu J. P. (2013). Herbal medicines for fatty liver diseases. Cochrane Database Syst. Rev. (8), CD009059. 10.1002/14651858.CD009059.pub2 23975682

[B73] Liu Q. Zhu L. Cheng C. Hu Y. Y. Feng Q. (2017). Natural Active Compounds from Plant Food and Chinese Herbal Medicine for Nonalcoholic Fatty Liver Disease. Curr. Pharm. Des. 23 (34), 5136–5162. 28925892 10.2174/1381612823666170918120643

[B74] Liu X. Tong W. Zhao X. Zhang H. Tang Y. Deng X. (2017). Chinese herb extract improves liver steatosis by promoting the expression of high molecular weight adiponectin in NAFLD rats. Mol. Med. Rep. 16 (4), 5580–5586. 10.3892/mmr.2017.7284 28849192

[B75] Liu Y. L. Lin L. C. Tung Y. T. Ho S. T. Chen Y. L. Lin C. C. (2017). Rhododendron oldhamii leaf extract improves fatty liver syndrome by increasing lipid oxidation and decreasing the lipogenesis pathway in mice. Int. J. Med. Sci. 14 (9), 862–870. 10.7150/ijms.19553 28824323 PMC5562193

[B76] Liu X. Xie Z. H. Liu C. Y. Zhang Y. (2019). Effect of Chinese Herbal Monomer Hairy Calycosin on Nonalcoholic Fatty Liver Rats and its Mechanism. Comb. Chem. High Throughput Screen 22 (3), 194–200. 10.2174/1386207322666190411112814 30973105

[B77] Liu Y. Y. You J. J. Xu W. Zhai T. Du C. Y. Chen Y. (2019). Gynura procumbens aqueous extract alleviates nonalcoholic steatohepatitis through CFLAR-JNK pathway in vivo and in vitro. Chin. Herb. Medicines 11 (4), 369–378. 10.1016/j.chmed.2019.09.005

[B78] Lo V. Erickson B. Thomason-Hughes M. Ko K. W. Dolinsky V. W. Nelson R. (2010). Arylacetamide deacetylase attenuates fatty-acid-induced triacylglycerol accumulation in rat hepatoma cells. J. Lipid Res. 51 (2), 368–377. 10.1194/jlr.M000596 19654421 PMC2803239

[B79] Lomonaco R. Sunny N. E. Bril F. Cusi K. (2013). Nonalcoholic fatty liver disease: current issues and novel treatment approaches. Drugs 73 (1), 1–14. 10.1007/s40265-012-0004-0 23329465

[B80] Lu X. Yuan Z. Y. Yan X. J. Lei F. Jiang J. F. Yu X. (2016). Effects of Angelica dahurica on obesity and fatty liver in mice. Chin. J. Nat. Med. 14 (9), 641–652. 10.1016/S1875-5364(16)30076-0 27667509

[B81] Luan H. Huo Z. Zhao Z. Zhang S. Huang Y. Shen Y. (2019). Scutellarin, a modulator of mTOR, attenuates hepatic insulin resistance by regulating hepatocyte lipid metabolism via SREBP-1c suppression. Phytother. Res. 10.1002/ptr.6582 31828866

[B82] Ma J. Zhao D. Wang X. Ma C. Feng K. Zhang S. (2019). LongShengZhi Capsule Reduces Established Atherosclerotic Lesions in apoE-Deficient Mice by Ameliorating Hepatic Lipid Metabolism and Inhibiting Inflammation. J. Cardiovasc. Pharmacol. 73 (2), 105–117. 10.1097/FJC.0000000000000642 30540683

[B83] Mandal S. Mukhopadhyay S. Bandhopadhyay S. Sen G. Biswas T. (2014). 14-Deoxyandrographolide alleviates ethanol-induced hepatosteatosis through stimulation of AMP-activated protein kinase activity in rats. Alcohol 48 (2), 123–132. 10.1016/j.alcohol.2013.11.005 24507479

[B84] Mato J. M. Alonso C. Noureddin M. Lu S. C. (2019). Biomarkers and subtypes of deranged lipid metabolism in non-alcoholic fatty liver disease. World J. Gastroenterol. 25 (24), 3009–3020. 10.3748/wjg.v25.i24.3009 31293337 PMC6603806

[B85] Meng Q. Duan X. P. Wang C. Y. Liu Z. H. Sun P. Y. Huo X. K. (2017). Alisol B 23-acetate protects against non-alcoholic steatohepatitis in mice via farnesoid X receptor activation. Acta Pharmacol. Sin. 38 (1), 69–79. 10.1038/aps.2016.119 27773935 PMC5220543

[B86] Moctezuma-Velazquez C. (2018). Current treatment for non-alcoholic fatty liver disease. Rev. Gastroenterol. Mex. 83 (2), 125–133. 10.1016/j.rgmxen.2018.05.014 29655574

[B87] Musso G. Gambino R. Cassader M. (2009). Recent insights into hepatic lipid metabolism in non-alcoholic fatty liver disease (NAFLD). Prog. Lipid Res. 48 (1), 1–26. 10.1016/j.plipres.2008.08.001 18824034

[B88] Nguyen P. Leray V. Diez M. Serisier S. Le Bloc’h J. Siliart B. (2008). Liver lipid metabolism. J. Anim. Physiol. Anim. Nutr. (Berl.) 92 (3), 272–283. 10.1111/j.1439-0396.2007.00752.x 18477307

[B89] Ouali F. Djouadi F. Merlet-Benichou C. Riveau B. Bastin J. (2000). Regulation of fatty acid transport protein and mitochondrial and peroxisomal beta-oxidation gene expression by fatty acids in developing rats. Pediatr. Res. 48 (5), 691–696. 10.1203/00006450-200011000-00023 11044493

[B90] Parafati M. Lascala A. Morittu V. M. Trimboli F. Rizzuto A. Brunelli E. (2015). Bergamot polyphenol fraction prevents nonalcoholic fatty liver disease via stimulation of lipophagy in cafeteria diet-induced rat model of metabolic syndrome. J. Nutr. Biochem. 26 (9), 938–948. 10.1016/j.jnutbio.2015.03.008 26025327

[B91] Park H. Kaushik V. K. Constant S. Prentki M. Przybytkowski E. Ruderman N. B. (2002). Coordinate regulation of malonyl-CoA decarboxylase, sn-glycerol-3-phosphate acyltransferase, and acetyl-CoA carboxylase by AMP-activated protein kinase in rat tissues in response to exercise. J. Biol. Chem. 277 (36), 32571–32577. 10.1074/jbc.M201692200 12065578

[B92] Park H. J. Han J. M. Kim H. G. Choi M. K. Lee J. S. Lee H. W. (2013). Chunggan extract (CGX), methionine-and choline-deficient (MCD) diet-induced hepatosteatosis and oxidative stress in C57BL/6 mice. Hum. Exp. Toxicol. 32 (12), 1258–1269. 10.1177/0960327113485253 23970447

[B93] Park H. Hwang Y. H. Kim D. G. Jeon J. Ma J. Y. (2015). Hepatoprotective effect of herb formula KIOM2012H against nonalcoholic fatty liver disease. Nutrients 7 (4), 2440–2455. 10.3390/nu7042440 25849950 PMC4425153

[B94] Park T. Y. Hong M. Sung H. Kim S. Suk K. T. (2017). Effect of Korean Red Ginseng in chronic liver disease. J. Ginseng Res. 41 (4), 450–455. 10.1016/j.jgr.2016.11.004 29021690 PMC5628344

[B95] Peng C. H. Liu L. K. Chuang C. M. Chyau C. C. Huang C. N. Wang C. J. (2011). Mulberry water extracts possess an anti-obesity effect and ability to inhibit hepatic lipogenesis and promote lipolysis. J. Agric. Food Chem. 59 (6), 2663–2671. 10.1021/jf1043508 21361295

[B96] Peng H. He Y. Zheng G. Zhang W. Yao Z. Xie W. (2016). Meta-analysis of traditional herbal medicine in the treatment of nonalcoholic fatty liver disease. Cell Mol. Biol. (Noisy-le-grand) 62 (4), 88–95. 27188741

[B97] Perla F. M. Prelati M. Lavorato M. Visicchio D. Anania C. (2017). The Role of Lipid and Lipoprotein Metabolism in Non-Alcoholic Fatty Liver Disease. Children (Basel) 4 (6). 10.3390/children4060046 PMC5483621 28587303

[B98] Perumpail B. J. Li A. A. Iqbal U. Sallam S. Shah N. D. Kwong W. (2018). Potential Therapeutic Benefits of Herbs and Supplements in Patients with NAFLD. Diseases 6 (3). 10.3390/diseases6030080 PMC6165515 30201879

[B99] Pierre G. Macdonald A. Gray G. Hendriksz C. Preece M. A. Chakrapani A. (2007). Prospective treatment in carnitine-acylcarnitine translocase deficiency. J. Inherit. Metab. Dis. 30 (5), 815. 10.1007/s10545-007-0518-x 17508264

[B100] Pil Hwang Y. Gyun Kim H. Choi J. H. Truong Do M. Tran T. P. Chun H. K. (2013). 3-Caffeoyl, 4-dihydrocaffeoylquinic acid from Salicornia herbacea attenuates high glucose-induced hepatic lipogenesis in human HepG2 cells through activation of the liver kinase B1 and silent information regulator T1/AMPK-dependent pathway. Mol. Nutr. Food Res. 57 (3), 471–482. 10.1002/mnfr.201200529 23349077

[B101] Ponziani F. R. Pecere S. Gasbarrini A. Ojetti V. (2015). Physiology and pathophysiology of liver lipid metabolism. Expert Rev. Gastroenterol. Hepatol. 9 (8), 1055–1067. 10.1586/17474124.2015.1056156 26070860

[B102] Qian W. Cai X. Zhang X. Wang Y. Qian Q. Hasegawa J. (2016). Effect of Daisaikoto on Expressions of SIRT1 and NF-kappaB of Diabetic Fatty Liver Rats Induced by High-Fat Diet and Streptozotocin. Yonago Acta Med. 59 (2), 149–158. 27493486 PMC4973021

[B103] Qiu P. Li X. Kong D. S. Li H. Z. Niu C. C. Pan S. H. (2015). Herbal SGR Formula Prevents Acute Ethanol-Induced Liver Steatosis via Inhibition of Lipogenesis and Enhancement Fatty Acid Oxidation in Mice. Evid. Based Complement Alternat. Med. 2015, 613584. 10.1155/2015/613584 26101535 PMC4458561

[B104] Quiroga A. D. Lehner R. (2018). Pharmacological intervention of liver triacylglycerol lipolysis: The good, the bad and the ugly. Biochem. Pharmacol. 155, 233–241. 10.1016/j.bcp.2018.07.005 30006193

[B105] Reddy J. K. Rao M. S. (2006). Lipid metabolism and liver inflammation. II. Fatty liver disease and fatty acid oxidation. Am. J. Physiol. Gastrointest Liver Physiol. 290 (5), G852–G858. 16603729 10.1152/ajpgi.00521.2005

[B106] Ren L. Sun D. Zhou X. Yang Y. Huang X. Li Y. (2019). Chronic treatment with the modified Longdan Xiegan Tang attenuates olanzapine-induced fatty liver in rats by regulating hepatic de novo lipogenesis and fatty acid beta-oxidation-associated gene expression mediated by SREBP-1c, PPAR-alpha and AMPK-alpha. J. Ethnopharmacol. 232, 176–187. 10.1016/j.jep.2018.12.034 30590197

[B107] Roh J. S. Lee H. Lim J. Kim J. Yang H. Yoon Y. (2017). Effect of Gangjihwan on hepatic steatosis and inflammation in high fat diet-fed mice. J. Ethnopharmacol. 206, 315–326. 10.1016/j.jep.2017.06.008 28602867

[B108] Rui L. (2014). Energy metabolism in the liver. Compr. Physiol. 4 (1), 177–197. 10.1002/cphy.c130024 24692138 PMC4050641

[B109] Saha A. K. Ruderman N. B. (2003). Malonyl-CoA and AMP-activated protein kinase: an expanding partnership. Mol. Cell Biochem. 253 (1-2), 65–70. 10.1023/A:1026053302036 14619957

[B110] Seo M. S. Hong S. W. Yeon S. H. Kim Y. M. Um K. A. Kim J. H. (2014). Magnolia officinalis attenuates free fatty acid-induced lipogenesis via AMPK phosphorylation in hepatocytes. J. Ethnopharmacol. 157, 140–148. 10.1016/j.jep.2014.09.031 25261688

[B111] Shang J. Chen L. L. Xiao F. X. Sun H. Ding H. C. Xiao H. (2008). Resveratrol improves non-alcoholic fatty liver disease by activating AMP-activated protein kinase. Acta Pharmacol. Sin. 29 (6), 698–706. 10.1111/j.1745-7254.2008.00807.x 18501116

[B112] Sheng X. Wang M. Lu M. Xi B. Sheng H. Zang Y. Q. (2011). Rhein ameliorates fatty liver disease through negative energy balance, hepatic lipogenic regulation, and immunomodulation in diet-induced obese mice. Am. J. Physiol. Endocrinol. Metab. 300 (5), E886–E893. 10.1152/ajpendo.00332.2010 21364120

[B113] Sheng D. Zhao S. Gao L. Zheng H. Liu W. Hou J. (2019). BabaoDan attenuates high-fat diet-induced non-alcoholic fatty liver disease via activation of AMPK signaling. Cell Biosci. 9, 77. 10.1186/s13578-019-0339-2 31548878 PMC6751621

[B114] Shi K. Q. Fan Y. C. Liu W. Y. Li L. F. Chen Y. P. Zheng M. H. (2012). Traditional Chinese medicines benefit to nonalcoholic fatty liver disease: a systematic review and meta-analysis. Mol. Biol. Rep. 39 (10), 9715–9722. 10.1007/s11033-012-1836-0 22718512

[B115] Shi L. J. Shi L. Song G. Y. Zhang H. F. Hu Z. J. Wang C. (2013). Oxymatrine attenuates hepatic steatosis in non-alcoholic fatty liver disease rats fed with high fructose diet through inhibition of sterol regulatory element binding transcription factor 1 (Srebf1) and activation of peroxisome proliferator activated receptor alpha (Pparalpha). Eur. J. Pharmacol. 714 (1-3), 89–95. 10.1016/j.ejphar.2013.06.013 23791610

[B116] Shi X. Sun R. M. Zhao Y. Fu R. Wang R. W. Zhao H. Y. (2018). Promotion of autophagosome-lysosome fusion via salvianolic acid A-mediated SIRT1 up-regulation ameliorates alcoholic liver disease. Rsc Adv. 8 (36), 20411–20422. 10.1039/C8RA00798E PMC9080827 35541657

[B117] Singh R. Cuervo A. M. (2012). Lipophagy: connecting autophagy and lipid metabolism. Int. J. Cell Biol. 2012, 282041. 10.1155/2012/282041 22536247 PMC3320019

[B118] Sun F. Xie M. L. Zhu L. J. Xue J. Gu Z. L. (2009). Inhibitory effect of osthole on alcohol-induced fatty liver in mice. Dig. Liver Dis. 41 (2), 127–133. 10.1016/j.dld.2008.01.011 18339590

[B119] Sun X. H. Zhang L. D. Wei W. (2019). A study on the mechanism of adipokine in non-alcoholic fatty liver in rats treated by four herbs decoction. Eur. J. Inflammation 17, 8. 10.1177/2058739219853970

[B120] Tessari P. Coracina A. Cosma A. Tiengo A. (2009). Hepatic lipid metabolism and non-alcoholic fatty liver disease. Nutr. Metab. Cardiovasc. Dis. 19 (4), 291–302. 10.1016/j.numecd.2008.12.015 19359149

[B121] Theodotou M. Fokianos K. Moniatis D. Kadlenic R. Chrysikou A. Aristotelous A. (2019). Effect of resveratrol on non-alcoholic fatty liver disease. Exp. Ther. Med. 18 (1), 559–565. 10.3892/etm.2019.7607 31316594 PMC6566048

[B122] Uen W. C. Shi Y. C. Choong C. Y. Tai C. J. (2018). Cordycepin suppressed lipid accumulation via regulating AMPK activity and mitochondrial fusion in hepatocytes. J. Food Biochem. 42 (5), 7. 10.1111/jfbc.12569

[B123] Veeramani C. Alsaif M. A. Al-Numair K. S. (2017). Lavatera critica, a green leafy vegetable, controls high fat diet induced hepatic lipid accumulation and oxidative stress through the regulation of lipogenesis and lipolysis genes. BioMed. Pharmacother. 96, 1349–1357. 10.1016/j.biopha.2017.11.072 29174039

[B124] Veeramani C. Alsaif M. A. Al-Numair K. S. (2018). Herbacetin, a flaxseed flavonoid, ameliorates high percent dietary fat induced insulin resistance and lipid accumulation through the regulation of hepatic lipid metabolizing and lipid-regulating enzymes. Chem. Biol. Interact. 288, 49–56. 10.1016/j.cbi.2018.04.009 29653099

[B125] Wanders R. J. Waterham H. R. Ferdinandusse S. (2015). Metabolic Interplay between Peroxisomes and Other Subcellular Organelles Including Mitochondria and the Endoplasmic Reticulum. Front. Cell Dev. Biol. 3, 83. 26858947 10.3389/fcell.2015.00083 PMC4729952

[B126] Wang Y. L. Liu L. J. Zhao W. H. Li J. X. (2015). Intervening TNF-alpha via PPARgamma with Gegenqinlian Decoction in Experimental Nonalcoholic Fatty Liver Disease. Evid. Based Complement Alternat. Med. 2015, 715638. 26221176 10.1155/2015/715638 PMC4499399

[B127] Wang Y. Viscarra J. Kim S. J. Sul H. S. (2015). Transcriptional regulation of hepatic lipogenesis. Nat. Rev. Mol. Cell Biol. 16 (11), 678–689. 10.1038/nrm4074 26490400 PMC4884795

[B128] Wang Y. P. Wat E. Koon C. M. Wong C. W. Cheung D. W. Leung P. C. (2016). The beneficial potential of polyphenol-enriched fraction from Erigerontis Herba on metabolic syndrome. J. Ethnopharmacol. 187, 94–103. 10.1016/j.jep.2016.04.040 27125589

[B129] Wang S. J. Chen Q. Liu M. Y. Yu H. Y. Xu J. Q. Wu J. Q. (2019). Regulation effects of rosemary (Rosmarinus officinalis Linn.) on hepatic lipid metabolism in OA induced NAFLD rats. Food Funct. 10 (11), 7356–7365. 31650134 10.1039/c9fo01677e

[B130] Ward C. Martinez-Lopez N. Otten E. G. Carroll B. Maetzel D. Singh R. (2016). Autophagy, lipophagy and lysosomal lipid storage disorders. Biochim. Biophys. Acta 1861 (4), 269–284. 10.1016/j.bbalip.2016.01.006 26778751

[B131] Wu W. Y. Wang Y. P. (2012). Pharmacological actions and therapeutic applications of Salvia miltiorrhiza depside salt and its active components. Acta Pharmacol. Sin. 33 (9), 1119–1130. 10.1038/aps.2012.126 22941285 PMC4003110

[B132] Xiao J. Fai So K. Liong E. C. Tipoe G. L. (2013). Recent advances in the herbal treatment of non-alcoholic Fatty liver disease. J. Tradit. Complement Med. 3 (2), 88–94. 10.4103/2225-4110.110411 24716162 PMC3924972

[B133] Xu L. Yin L. Tao X. Qi Y. Han X. Xu Y. (2017). Dioscin, a potent ITGA5 inhibitor, reduces the synthesis of collagen against liver fibrosis: Insights from SILAC-based proteomics analysis. Food Chem. Toxicol. 107 (Pt A), 318–328. 10.1016/j.fct.2017.07.014 28689917

[B134] Yan S. Khambu B. Hong H. Liu G. Huda N. Yin X. M. (2019). Autophagy, Metabolism, and Alcohol-Related Liver Disease: Novel Modulators and Functions. Int. J. Mol. Sci. 20 (20). 10.3390/ijms20205029 PMC6834312 31614437

[B135] Yang W. She L. Yu K. Yan S. Zhang X. Tian X. (2016). Jatrorrhizine hydrochloride attenuates hyperlipidemia in a high-fat diet-induced obesity mouse model. Mol. Med. Rep. 14 (4), 3277–3284. 10.3892/mmr.2016.5634 27573054

[B136] Yang L. Lin W. Nugent C. A. Hao S. Song H. Liu T. (2017). Lingguizhugan Decoction Protects against High-Fat-Diet-Induced Nonalcoholic Fatty Liver Disease by Alleviating Oxidative Stress and Activating Cholesterol Secretion. Int. J. Genomics 2017, 2790864. 10.1155/2017/2790864 29464180 PMC5804362

[B137] Yang J. M. Sun Y. Wang M. Zhang X. L. Zhang S. J. Gao Y. S. (2019). Regulatory effect of a Chinese herbal medicine formula on non-alcoholic fatty liver disease. World J. Gastroenterol. 25 (34), 5105–5119. 10.3748/wjg.v25.i34.5105 31558860 PMC6747291

[B138] Yang L. Yang C. Thomes P. G. Kharbanda K. K. Casey C. A. McNiven M. A. (2019). Lipophagy and Alcohol-Induced Fatty Liver. Front. Pharmacol. 10, 495. 10.3389/fphar.2019.00495 31143122 PMC6521574

[B139] Yang X. X. Wang X. Shi T. T. Dong J. C. Li F. J. Zeng L. X. (2019). Mitochondrial dysfunction in high-fat diet-induced nonalcoholic fatty liver disease: The alleviating effect and its mechanism of Polygonatum kingianum. BioMed. Pharmacother. 117, 109083. 10.1016/j.biopha.2019.109083 31387169

[B140] Yang Y. Li J. Wei C. He Y. Cao Y. Zhang Y. (2019). Amelioration of nonalcoholic fatty liver disease by swertiamarin in fructose-fed mice. Phytomedicine 59, 152782. 10.1016/j.phymed.2018.12.005 31005808

[B141] Yao H. Qiao Y. J. Zhao Y. L. Tao X. F. Xu L. N. Yin L. H. (2016). Herbal medicines and nonalcoholic fatty liver disease. World J. Gastroenterol. 22 (30), 6890–6905. 10.3748/wjg.v22.i30.6890 27570425 PMC4974587

[B142] Yoon S. Kim J. Lee H. Lee H. Lim J. Yang H. (2017). The effects of herbal composition Gambigyeongsinhwan (4) on hepatic steatosis and inflammation in Otsuka Long-Evans Tokushima fatty rats and HepG2 cells. J. Ethnopharmacol. 195, 204–213. 10.1016/j.jep.2016.11.020 27845265

[B143] Younossi Z. M. (2019). Non-alcoholic fatty liver disease - A global public health perspective. J. Hepatol. 70 (3), 531–544. 10.1016/j.jhep.2018.10.033 30414863

[B144] Yu S. Rao S. Reddy J. K. (2003). Peroxisome proliferator-activated receptors, fatty acid oxidation, steatohepatitis and hepatocarcinogenesis. Curr. Mol. Med. 3 (6), 561–572. 10.2174/1566524033479537 14527087

[B145] Zamani N. Shams M. Nimrouzi M. Zarshenas M. M. Abolhasani Foroughi A. Fallahzadeh Abarghooei E. (2018). The effects of Zataria multiflora Boiss. (Shirazi thyme) on nonalcoholic fatty liver disease and insulin resistance: A randomized double-blind placebo-controlled clinical trial. Complement Ther. Med. 41, 118–123. 10.1016/j.ctim.2018.09.010 30477827

[B146] Zar Kalai F. Han J. Ksouri R. Abdelly C. Isoda H. (2014). Oral administration of Nitraria retusa ethanolic extract enhances hepatic lipid metabolism in db/db mice model ‘BKS.Cg-Dock7(m)+/+ Lepr(db/)J’ through the modulation of lipogenesis-lipolysis balance. Food Chem. Toxicol. 72, 247–256. 10.1016/j.fct.2014.07.029 25086370

[B147] Zhang Y. Si Y. Zhai L. Yang N. Yao S. Sang H. (2013). Celastrus orbiculatus Thunb. ameliorates high-fat diet-induced non-alcoholic fatty liver disease in guinea pigs. Pharmazie 68 (10), 850–854. 24273892

[B148] Zhang Y. Yu L. Cai W. Fan S. Feng L. Ji G. (2014). Protopanaxatriol, a novel PPARgamma antagonist from Panax ginseng, alleviates steatosis in mice. Sci. Rep. 4, 7375. 10.1038/srep07375 25487878 PMC4260220

[B149] Zhang E. Yin S. Song X. Fan L. Hu H. (2016). Glycycoumarin inhibits hepatocyte lipoapoptosis through activation of autophagy and inhibition of ER stress/GSK-3-mediated mitochondrial pathway. Sci. Rep. 6, 38138. 10.1038/srep38138 27901086 PMC5128870

[B150] Zhang L. Yao Z. Ji G. (2018). Herbal Extracts and Natural Products in Alleviating Non-alcoholic Fatty Liver Disease via Activating Autophagy. Front. Pharmacol. 9, 1459. 10.3389/fphar.2018.01459 30618753 PMC6297257

[B151] Zhang E. Yin S. Zhao S. Zhao C. Yan M. Fan L. (2019). Protective effects of glycycoumarin on liver diseases. Phytother. Res. 10.1002/ptr.6598 31840883

[B152] Zhang Y. Liu M. Chen Q. Wang T. Yu H. Xu J. (2019). Leaves of Lippia triphylla improve hepatic lipid metabolism via activating AMPK to regulate lipid synthesis and degradation. J. Nat. Med. 73 (4), 707–716. 10.1007/s11418-019-01316-5 31104252

[B153] Zheng Y. Wang M. Zheng P. Tang X. Ji G. (2018). Systems pharmacology-based exploration reveals mechanisms of anti-steatotic effects of Jiang Zhi Granule on non-alcoholic fatty liver disease. Sci. Rep. 8 (1), 13681. 30209324 10.1038/s41598-018-31708-8 PMC6135841

[B154] Zhong H. Chen K. Feng M. Shao W. Wu J. Chen K. (2018). Genipin alleviates high-fat diet-induced hyperlipidemia and hepatic lipid accumulation in mice via miR-142a-5p/SREBP-1c axis. FEBS J. 285 (3), 501–517. 10.1111/febs.14349 29197188

[B155] Zhou W. Rahimnejad S. Lu K. Wang L. Liu W. (2019). Effects of berberine on growth, liver histology, and expression of lipid-related genes in blunt snout bream (Megalobrama amblycephala) fed high-fat diets. Fish Physiol. Biochem. 45 (1), 83–91. 10.1007/s10695-018-0536-7 29984398

[B156] Zhu M. Hao S. Liu T. Yang L. Zheng P. Zhang L. (2017). Lingguizhugan decoction improves non-alcoholic fatty liver disease by altering insulin resistance and lipid metabolism related genes: a whole trancriptome study by RNA-Seq. Oncotarget 8 (47), 82621–82631. 10.18632/oncotarget.19734 29137289 PMC5669915

